# Supplementary material for: Heterotypic interactions can drive selective co-condensation of prion-like low-complexity domains of FET proteins and mammalian SWI/SNF complex
Source: Nat Commun. 2024 Feb 7;15:1168. doi: 10.1038/s41467-024-44945-5 (PMC10850361; doi:10.1038/s41467-024-44945-5)
Supplement: Supplementary file 1 — Supplementary Information File [file 41467_2024_44945_MOESM1_ESM.pdf]

Supplementary Information for

Heterotypic interactions can drive selective co-condensation of prion-like low-complexity domains of FET proteins and mammalian SWI/SNF complex

Authors: Richoo B. Davis[1], Anushka Supakar[2], Aishwarya Kanchi Ranganath[3], Mahdi Muhammad Moosa[1], Priya R. Banerjee[1, 2,3] *

Affiliations:

[1]Department of Physics, University at Buffalo, Buffalo NY 14260, USA

[2]Department of Biological Sciences, University at Buffalo, Buffalo NY 14260, USA

[3]Department of Chemical and Biological Engineering, University at Buffalo, Buffalo NY 14260, USA

*Corresponding Author: P.R.B. prbanerj@buffalo.edu

Supplementary Tables

	ARID1A^{PLD}	ARID1B^{PLD}	BRG1^{PLD}	SS18^{PLD}	SMARCC1^{PLD}	FUS^{PLD}	Pol II CTD^{30}	EWSR1^{PLD}	TAF15^{PLD}
Ala (A)	12	14.9	7.4	0.9	8	2.3	0.5	10.2	0.5
Arg (R)	2.3	2.7	2.6	2	1.3	0	0.5	0.4	4.9
Asn (N)	2.7	2	2.4	7.1	2.7	2.9	2.4	1.5	5.9
Asp (D)	1.3	1.2	2.4	2.6	0.7	1.2	0	1.9	7.4
Cys (C)	0.2	0.7	0	0	0	0.6	0	0	0
Gln (Q)	16.8	12.1	7.9	20.5	12	21.4	0.5	17.7	17.6
Glu (E)	0.6	0.5	1.2	1.4	0.7	0	0.5	0.4	2.9
Gly (G)	15.8	22.8	16.5	16.5	13.3	20.2	0.9	9.4	21.1
His (H)	2.5	2	3.2	3.1	6.7	0	0	0	2.5
Ile (I)	0.2	0.5	0.9	0.3	2.7	0	0	0	0
Leu (L)	2.9	2.5	3.5	0.9	3.3	0	0	0.4	0
Lys (K)	0.8	0.7	2.1	0	0	0	0	0.4	1
Met (M)	1.9	5.4	7.6	8.8	8	0.6	0.5	0.8	0.5
Phe (F)	1.1	0.7	0.3	0	0	0	0	0	0
Pro (P)	16.6	12.6	26.5	17.9	32	6.4	28.3	11.3	1.5
Ser (S)	12.6	10.4	9.1	6.8	3.3	25.4	37.3	15.1	17.6
Thr (T)	3.4	1.5	2.9	1.1	2	5.2	14.6	14.7	2.9
Trp (W)	0.2	0.2	0.3	0	0	0	0	0	0
Tyr (Y)	5.5	5.4	1.5	9.1	1.3	13.9	14.2	14	13.2
Val (V)	0.6	1	1.8	1.1	2	0	0	1.9	0.5

Table S1: Amino acid composition (in percentage) for each of the prion-like domains (PLDs) used in this study. The red color intensity scales linearly with the percentage of a given amino acid.

	ARID1A^{PLD}	ARID1B^{PLD}	BRG1^{PLD}	SS18^{PLD}	SMARCC1^{PLD}	FUS^{PLD}	Pol II CTD^{30}	EWSR1^{PLD}	TAF15^{PLD}
Ala (A)	63	60	25	3	12	4	1	27	1
Arg (R)	12	11	9	7	2	0	1	1	10
Asn (N)	14	8	8	25	4	5	5	4	12
Asp (D)	7	5	8	9	1	2	0	5	15
Cys (C)	1	3	0	0	0	1	0	0	0
Gln (Q)	88	49	27	72	18	37	1	47	36
Glu (E)	3	2	4	5	1	0	1	1	6
Gly (G)	83	92	56	58	20	35	2	25	43
His (H)	13	8	11	11	10	0	0	0	5
Ile (I)	1	2	3	1	4	0	0	0	0
Leu (L)	15	10	12	3	5	0	0	1	0
Lys (K)	4	3	7	0	0	0	0	1	2
Met (M)	10	22	26	31	12	1	1	2	1
Phe (F)	6	3	1	0	0	0	0	0	0
Pro (P)	87	51	90	63	48	11	60	30	3
Ser (S)	66	42	31	24	5	44	79	40	36
Thr (T)	18	6	10	4	3	9	31	39	6
Trp (W)	1	1	1	0	0	0	0	0	0
Tyr (Y)	29	22	5	32	2	24	30	37	27
Val (V)	3	4	6	4	3	0	0	5	1

Table S2: Number of amino acids in each of the prion-like domains (PLDs) used in this study. The red color intensity scales linearly with the number of a given amino acid.

	ARID1A^{PLD}	ARID1B^{PLD}	BRG1^{PLD}	SS18^{PLD}	SMARCC1^{PLD}	FUS^{PLD}	Pol II CTD^{30}	EWSR1^{PLD}	TAF15^{PLD}
Aromatic stickers (FYW)	36	26	7	32	2	24	30	37	27
Positive Charges (RK)	16	14	16	7	2	0	1	2	12
Negative charges (DE)	10	7	12	14	2	2	1	6	21
Hydrophobic (ILMV)	29	38	47	39	24	1	1	8	2
Arginines +Aromatic (R+FYW)	48	37	16	39	4	24	31	38	10
NCPR	0.011	0.017	0.012	-0.020	0.000	-0.011	0.000	-0.015	-0.044
Length	523	403	340	352	150	173	212	265	204
Sticker density ((R+FYW)/ Length)	0.09	0.09	0.05	0.11	0.03	0.14	0.15	0.14	0.18

Table S3: Amino acid features of the prion-like domains employed in this work. Net charge per residue (NCPR) values were obtained using the CIDER tool [1]. The red color intensity scales linearly with the value of a given parameter.

Protein	Purpose	Sequence
BRG1^{PLD}	Purification/ live-cell imaging	MSTPDPPLGGTPRPGPSPGPGPSPGAMLGPSPGPSPGSAHSMMGPS<br>PGPPSAGHPIPTQGPGGYPQDNMHQMHKPMESMHEKGMSDDPRYNQ<br>MKGMGMRSGGHAGMGPPPSPMDQHSQGYPSPLGGSEHASSPVPAS<br>GPSSGPQMSSGPGGAPLDGADPQALGQQNRGPTPFNQNQLHQLRAQI<br>MAYKMLARGQPLPDHLQMAVQGKRPMPGMQQQMPTLPPPSVSATGP<br>GPGPGPGPGPGPGPAPPNYSRPHGMGGPNMPPPGPSGVPPGMPGQ<br>PPGGPPKPWPEGPMANAAAPTSTPQKLIPPQPTGRPSPAPPAVPPAAS<br>PVMPPQTQSPGQPAQPA
BRG1^{PLD} 2C	Purification/ Cys-maleimide conjugation for site-specific protein labeling	MCSTPDPPLGGTPRPGPSPGPGPSPGAMLGPSPGPSPGSAHSMMGP<br>SPGPPSAGHPIPTQGPGGYPQDNMHQMHKPMESMHEKGMSDDPRYN<br>QMKGMGMRSGGHAGMGPPPSPMDQHSQGYPSPLGGSEHASSPVPA<br>SGPSSGPQMSSGPGGAPLDGADPQALGQQNRGPTPFNQNQLHQLRA<br>QIMAYKMLARGQPLPDHLQMAVQGKRPMPGMQQQMPTLPPPSVSATG<br>PGPGPGPGPGPGPGPAPPNYSRPHGMGGPNMPPPGPSGVPPGMPG<br>QPPGGPPKPWPEGPMANAAAPTSTPQKLIPPQPTGRPSPAPPAVPPAA<br>SPVMPPQTQSPGQPAQPA
ARID1A^{PLD}	Purification/ Cys-maleimide conjugation for site-specific protein labeling / live-cell imaging	MSNGGGGGGGAGSGGGPGAEPDLKNSNGNAGPRPALNNNLTEPPGG<br>GGGGSSDGVGAPPHSAAAALPPPAYGFGQPYGRSPSAVAAAAAAVFH<br>QQHGGQQSPGLAALQSGGGGGLEPYAGPQQNSHDHGFPNHQYNSYY<br>PNRSAYPPPAPAYALSSPRGGTPGSGAAAAAGSKPPPSSSASASSSSS<br>SFAQQRFGAMGGGGPSAAGGGTPQPTATPTLNQLLTSPSSARGYQGY<br>PGGDYSGGPQDGGAGKGPADMASQCWGAAAAAAAAAAAASGGAQQR<br>SHHAPMSPGSSGGGGQPLARTPQPSSPMDQMGKMRPQPYGGTNPYS<br>QQQGPPSGPQQGHGYPGQPYGSQTPQRYPMTMQGRAQSAMGGLSY<br>TQQIPPYGQQGPSGYGQQGQTPYYNQQSPHPQQQQPPYSQQPPSQT<br>PHAQPSYQQQPQSQPPQLQSSQPPYSQQPSQPPHQQSPAPYPSQQS<br>TTQQHPQSQPPYSQPQAQSPYQQQQPQQPAPSTLSQQAAYPQPQSQ<br>QSQQTAYSQQRFPPPQ

ARID1B^{PLD}	Purification/ Cys-maleimide conjugation for site-specific protein labeling / live-cell imaging	MNNYYGSAAPASGGPGGRAGPCFDQHGGQQSPGMGMMHSASAAAA GAPGSMDPLQNSHEGYPNSQCNHYPGYSRPGAGGGGGGGGGGGGG SGGGGGGGGAGAGGAGAGAVAAAAAAAAAAAAGGGGGGGYGGSSAG YGVLSSPRQQGGGMMMGPGGGGAASLSKAAAGSAAGGFQRFAGQN QHPSGATPTLNQLLTSPSPMMRSYGGSYPEYSSPSAPPPPPSQPQSQA AAAGAAAGGQQAAAGMGLGKDMGAQYAAASPAWAAAQQRSHPAMSP GTPGPTMGRSQGSPMDPMVMKRPQLYGMGSNPHSQPQQSSPYPGG SYGPPGPQRYPIGIQGRTPGAMAGMQYPQQQMPPQYGQQGVSGYCQ QGQQPYYSQQPQPPHLPPQAQYLPSQSQQRYQPQQDMSQ
SS18^{PLD}	Purification/ live-cell imaging	MNQNMQSLLPAPPTQNMPMGPGGMNQSGPPPPPRSHNMPSDGMVG GGPPAPHMQNQMNGQMPGPNHMPMQGPGPNQLNMTNSSMNMPSSS HGSMGGYNHSVPSSQSMPVQNQMTMSQGQPMGNYGPRPNMSMQPN QGPMMHQQPPSQQYNMPQGGGQHYQGQQPPMGMMGQVNQGNHM MGQRQIPPYRPPQQGPPQQYSGQEDYYGDQYSHGGQGPPEGMNQQ YYPDGHNDYGYQQPSYPEQGYDRPYEDSSQHYYEGGNSQYGQQQDA YQGPPPQQGYPPQQQQYPGQQGYPGQQQGYGPSQGGPGPQYPNYP QGQGQQYGGYRPTQPGPPQPPQQRPYGYDQGQYGNYQQ
SS18^{PLD} 2C	Purification/ Cys-maleimide conjugation for site-specific protein labeling	MCNQNMQSLLPAPPTQNMPMGPGGMNQSGPPPPPRSHNMPSDGMV GGGPPAPHMQNQMNGQMPGPNHMPMQGPGPNQLNMTNSSMNMPS SSHGSMGGYNHSVPSSQSMPVQNQMTMSQGQPMGNYGPRPNMSMQ PNQGPMMHQQPPSQQYNMPQGGGQHYQGQQPPMGMMGQVNQGNH MMGQRQIPPYRPPQQGPPQQYSGQEDYYGDQYSHGGQGPPEGMNQ QYYPDGHNDYGYQQPSYPEQGYDRPYEDSSQHYYEGGNSQYGQQQD AYQGPPPQQGYPPQQQQYPGQQGYPGQQQGYGPSQGGPGPQYPNY PQGQGQQYGGYRPTQPGPPQPPQQRPYGYDQGQYGNYQQ
SMARCC1^{PLD}	Live cell imaging	QQMEQQQHGQNPQQAHQHSGGPGLAPLGAAGHPGMMPHQQPPPYP LMHHQMPPPHPPQPGQIPGPGSMMPGQHMPGRMIPTVAANIHPSGSG PTPPGMPPMPGNILGPRVPLTAPNGMYPPPPQQQPPPPPPADGVPPPP APGPPASAAP
FUS^{PLD}	Purification/ live-cell imaging/ optogenetic constructs	MASNDYTQQATQSYGAYPTQPGQGYSQQSSQPYGQQSYSGYSQSTD TSGYGQSSYSSYGQSQNSYGTQSTPQGYGSTGGYGSSQSSQSSYGQ QSSYPGYGQQPAPSSTSGSYGSSSQSSSYGQPQSGSYSQQPSYGGQ QQSYGQQQSYNPPQGYGQQNQYNSSSGGGGGGGGG
FUS^{PLD} S86C	Purification/ Cys-maleimide conjugation for site-specific protein labeling	MASNDYTQQATQSYGAYPTQPGQGYSQQSSQPYGQQSYSGYSQSTD TSGYGQSSYSSYGQSQNSYGTQSTPQGYGSTGGYGSSQSCQSSYGQ

		QSSYPGYGQQPAPSSTSGSYGSSSQSSSYGQPQSGSYSQQPSYGGQ QQSYGQQQSYNPPQGYGQQNQYNSSSGGGGGGGGG
EWSR1^PLD	Live cell imaging	MASTDYSTYSQAAAQQGYSAYTAQPTQGYAQTTQAYGQQSYGTYGQP TDVSYTQAQTTATYGQTAYATSYGQPPTGYTTPTAPQAYSQPVQGYGT GAYDTTTATVTTTQASYAAQSAYGTQPAYPAYGQQPAATAPTRPQDGN KPTETSQPQSSTGGYNQPSLGYGQSNYSYPQVPGSYPMQPVTAPPSY PPTSYSSTQPTSYDQSSYSQQNTYGQPSSYGQQSSYGQQSSYGQQPP TSYPPQTGSYSQAPSQYSQQSSSYGQQS
TAF15^PLD	Live cell imaging	SDSGSYSQSGGEQQSYSSYGNQGSQGYGQTPQGYSGYGQTTDSSYG QNYGGYSGYGQNQSGYSQSYGSYENQKQSSYGQQSYNNQGQQNTE SSGGQGGRAPSYGQSDYGQQDSYDQQSGYDQHQGSYDEQSNYQQH DSYNQNQQSYHSQRENYSHHTQDDRRDVSRYGEDNRGYGGSQGGG RGRGGYDKDGRGPMTGSSGGDRG
RNA Pol II CTD^30	Live cell imaging	YSPTSPAYEPRSPGGYTPQSPSYSPTSPSYSPTSPSYSPTSPNYSPTSP SYSPTSPSYSPTSPSYSPTSPSYSPTSPSYSPTSPSYSPTSPSYSPTSP SYSPTSPSYSPTSPSYSPTSPSYSPTSPSYSPTSPSYSPTSPSYSPTSP SYSPTSPNYSPTSPNYTPTSPSYSPTSPSYSPTSPNYTPTSPNYSPTSP SYSPTSPSYSPTSPS
RNA Pol II CTD^30 2C	Purification/ Cys-maleimide conjugation for site-specific protein labeling	MCYSPTSPAYEPRSPGGYTPQSPSYSPTSPSYSPTSPSYSPTSPNYSP TSPSYSPTSPSYSPTSPSYSPTSPSYSPTSPSYSPTSPSYSPTSPSYSPT SPSYSPTSPSYSPTSPSYSPTSPSYSPTSPSYSPTSPSYSPTSPSYSPT SPSYSPTSPNYSPTSPNYTPTSPSYSPTSPSYSPTSPNYTPTSPNYSPT SPSYSPTSPSYSPTSPS
FOXG1^N-IDR	Live cell imaging	MLDMGDRKEVKMIPKSSFSINSLVPEAVQN DNHHASHGHHNSHHPQH HHHHHHHHHHPPPPAPQPPPPPQQQQPPPPPPPPAPQPPQTRGAPAAD DDKGPQQLLLPPPPPPPPAAALDGAKADGLGGKGEPGGGPGELAPVG PDEKEKGAGAGGEEKKGAGEGGKDGEGGKEGEKKNGKYE
FOXG1^N-IDR 2C	Purification/ Cys-maleimide conjugation for site-specific protein labeling /	MCLDMGDRKEVKMIPKSSFSINSLVPEAVQN DNHHASHGHHNSHHPQ HHHHHHHHHHHPPPPAPQPPPPPQQQQPPPPPPPPAPQPPQTRGAPAA DDDKGPQQLLLPPPPPPPPAAALDGAKADGLGGKGEPGGGPGELAPV GPDEKEKGAGAGGEEKKGAGEGGKDGEGGKEGEKKNGKYE
His-MBP-TEV-cys	Purification/ Cys-maleimide conjugation for site-specific protein labeling	GGGCGGG

BRG1$^{\text{PLD Aro+}}$	Live cell imaging	MSTPDPPLGGTYRPGPSPGPGPSPGAMLGPSPGPSYGSAHSMMGPS<br>PGPYSAGHPIPTQGPGGYYQDNMHQMHKPMESMHEKGMSDDPRYNQ<br>MKGMGMRSGGHAGMGPYPSPMDQHSQGYPSPLGGSEHASSPVPAS<br>GPSSGPQMSSGPGGAYLDGADPQALGQQNRGPTPFNQNQLHQLRAQI<br>MAYKMLARGQYLPDHLQMAVQGKRPMPGMQQQMYTLPPPSVSATGY<br>GPGPGPGPGPGYGPAPPNYSRYHGMGGPNMPPYGPSGVPPGMPGQ<br>PPGGPPKPWYEGPMANAAAPTSTPQKLIYPQPTGRPSYAPPAVPYAAS<br>PVMPPQTQSYGQPAQPA
BRG1$^{\text{PLD Aro++}}$	Live cell imaging	MSTPDYPLGGTYRPGYSPGYGPSYGAMLGPSPGPSYGSAHSMMGPS<br>YGPYSAGHPIYTQGPGGYYQDNMHQMHKPMESMHEKGMSDDYRYNQ<br>MKGMGMRSGGHAGMGPYPSYMDQHSQGYPSYLGGSEHASSPVPAS<br>GPSSGYQMSSGPGGAYLDGADPQALGQQNRGYTPFNQNQLHQLRAQI<br>MAYKMLARGQYLPDHLQMAVQGKRYMPGMQQQMYTLPYPSVSATGY<br>GPGPGPGYGPGYGPAYPNYSRYHGMGGPNMYPYGPSGVYPGMYGQ<br>PYGGPPKPWYEGPMANAAAYTSTPQKLIYPQYTGRPSYAPYAVPYAAS<br>PVMYPQTQSYGQPAQYA
FUS$^{\text{2XPLD}}$	Live cell imaging	MASNDYTQQATQSYGAYPTQPGQGYSQQSSQPYGQQSYSGYSQSTD<br>TSGYGQSSYSSYGQSQNSYGTQSTPQGYGSTGGYGSSQSSQSSYGQ<br>QSSYPGYGQQPAPSSTSGSYGSSSQSSSYGQPQSGSYSQQPSYGGQ<br>QQSYGQQQSYNPPQGYGQQNQYNSSSGGGGGGGGGMASNDYTQQA<br>TQSYGAYPTQPGQGYSQQSSQPYGQQSYSGYSQSTDTSGYGQSSYS<br>SYGQSQNSYGTQSTPQGYGSTGGYGSSQSSQSSYGQQSSYPGYGQQ<br>PAPSSTSGSYGSSSQSSSYGQPQSGSYSQQPSYGGQQQSYGQQQSY<br>NPPQGYGQQNQYNSSSGGGGGGGGG
FUS$^{\text{2XPLD halfYtoS}}$	Live cell imaging	MASNDYTQQATQSYGAYPTQPGQGYSQQSSQPYGQQSYSGYSQSTD<br>TSGYGQSSYSSYGQSQNSYGTQSTPQGYGSTGGYGSSQSSQSSYGQ<br>QSSYPGYGQQPAPSSTSGSYGSSSQSSSYGQPQSGSYSQQPSYGGQ<br>QQSYGQQQSYNPPQGYGQQNQYNSSSGGGGGGGGGMASNDSTQQA<br>TQSSGASPTQPGQGSSQQSSQPSGQQSSSGSSQSTDTSGSGQSSSS<br>SSGQSQNSSGTQSTPQGSGSTGGSGSSQSSQSSSGQQSSSPGSGQQ<br>PAPSSTSGSSGSSSQSSSSGQPQSGSSSQQPSSGGQQQSSGQQQSS<br>NPPQGSGQQNQSNSSSGGGGGGGGG
BRG1^{Folded}	Live cell imaging	EYGVSQALARGLQSYYAVAHAVTERVDKQSALMVNGVLKQYQIKGLEW<br>LVSLYNNNLNGILADEMGLGKTIQTIALITYLMEHKRINGPFLIIVPLSTLSN

		WAYEFDKWAPSVVKVSYKGSPAARRAFVPQLRSGKFNVLLTTYEYIIKD KHILAKIRWKYMIVDEGHRMKNHHCKLTQVLNTHYVAPRRLLLTGTPLQ NKLPELWALLNFLLPTIFKSCSTFEQWFNAPFAMTGEKVDLNEEETILIIR RLHKVLRPFLLRRLKKEVEAQLPEKVEYVIKCDMSALQRVLYRHMQAKG VLLTDGSEKDKKGKGGTKTLMNTIMQLRKICNHPYMFQHIEESFSEHLG FTGGIVQGLDLYRASGKFELLDRILPKLRATNHKVLLFCQMTSLMTIMED YFAYRGFKYLRLDGTTKAEDRGMLLKTFNEPGSEYFIFLLSTRAGGLGL NLQSADTVIIFDSDWNPHQDLQAQDRAHRIGQQNEVRVLRLCTVNSVEE KILAAAKYKLNVDQKVIQAGM
BRG1	Live-cell imaging	MSTPDPPLGGTPRPGPSPGPGPSPGAMLGPSPGPSPGSAHSMMGPS PGPPSAGHPIPTQGPGGYPQDNMHQMHKPMESMHEKGMSDDPRYNQ MKGMGMRSGGHAGMGPPPSPMDQHSQGYPSPLGGSEHASSPVPAS GPSSGPQMSSGPGGAPLDGADPQALGQQNRGPTPFNQNQLHQLRAQI MAYKMLARGQPLPDHLQMAVQGKRPMPGMQQQMPTLPPPSVSATGP GPGPGPGPGPGPAPPNYSRPHGMGGPNMPPPGPSGVPPGMPGQ PPGGPPKPWPEGPMANAAAPTSTPQKLIPPQPTGRPSPAPPAVPPAAS PVMPPQTQSPGQPAQPAPMVPLHQKQSRITPIQKPRGLDPVEILQEREY RLQARIAHRIQELENLPGSLAGDLRTKATIELKALRLLNFQRQLRQEVVV CMRRDTALETALNAKAYKRSKRQSLREARITEKLEKQQKIEQERKRRQK HQEYLNSILQHAKDFKEYHRSVTGKIQKLTKAVATYHANTEREQKKENE RIEKERMRRLMAEDEEGYRKLIDQKKDKRLAYLLQQTDEYVANLTELVR QHKAAQVAKEKKKKKKKKKAENAEGQTPAIGPDGEPLDETSQMSDLPV KVIHVESGKILTGTDAPKAGQLEAWLEMNPGYEVAPRSDSEESGSEEEE EEEEEEQPQAAQPPTLPVEEKKKIPDPDSDDVSEVDARHIIENAKQDVD DEYGVSQALARGLQSYYAVAHAVTERVDKQSALMVNGVLKQYQIKGLE WLVSLYNNNLNGILADEMGLGKTIQTIALITYLMEHKRINGPFLIIVPLSTLS NWAYEFDKWAPSVVKVSYKGSPAARRAFVPQLRSGKFNVLLTTYEYIIK DKHILAKIRWKYMIVDEGHRMKNHHCKLTQVLNTHYVAPRRLLLTGTPL QNKLPELWALLNFLLPTIFKSCSTFEQWFNAPFAMTGEKVDLNEEETILII RRLHKVLRPFLLRRLKKEVEAQLPEKVEYVIKCDMSALQRVLYRHMQAK GVLLTDGSEKDKKGKGGTKTLMNTIMQLRKICNHPYMFQHIEESFSEHL GFTGGIVQGLDLYRASGKFELLDRILPKLRATNHKVLLFCQMTSLMTIME DYFAYRGFKYLRLDGTTKAEDRGMLLKTFNEPGSEYFIFLLSTRAGGLG LNLQSADTVIIFDSDWNPHQDLQAQDRAHRIGQQNEVRVLRLCTVNSVE EKILAAAKYKLNVDQKVIQAGMFDQKSSSHERRAFLQAILEHEEQDESR HCSTGSGSASFAHTAPPPAGVNPDLEEPPLKEEDEVPDDETVNQMIAR

		HEEEFDLFMRMDLDRRREEARNPKRKPRLMEEDELPSWIIKDDAEVERL<br>TCEEEEEKMFGRGSRHRKEVDYSDSLTEKQWLKAIEEGTLEEIEEEVRQ<br>KKSSRKRKRDSDAGSSTPTTSTRSRDKDDESKKQKKRGRPPAEKLSPN<br>PPNLTKKMKKIVDAVIKYKDSSSGRQLSEVFIQLPSRKELPEYYELIRKPV<br>DFKKIKERIRNHKYRSLNDLEKDVMLLCQNAQTFNLEGSLIYEDSIVLQSV<br>FTSVRQKIEKEDDSEGEESEEEEEGEEEGSESESRSVKVKIKLGRKEKA<br>QDRLKGGRRRPSRGSRAKPVVSDDDSEEEQEEDRSGSGSEED
ARID1A$^{\text{PLD 30QtoG}}$	Live cell imaging	MSNGGGGGGGAGSGGGPGAEPDLKNSNGNAGPRPALNNNLTEPPGG<br>GGGGSSDGVGAPPHSAAAALPPPAYGFGQPYGRSPSAVAAAAAAVFH<br>QQHGGQQSPGLAALQSGGGGGLEPYAGPQQNSHDHGFPNHQYNSYY<br>PNRSAYPPPAPAYALSSPRGGTPGSGAAAAAGSKPPPSSSASASSSSS<br>SFAQQRFGAMGGGGPSAAGGGTPQPTATPTLNQLLTSPSSARGYQGY<br>PGGDYSGGPQDGGAGKGPADMASQCWGAAAAAAAAAAASGGAQQR<br>SHHAPMSPGSSGGGGQPLARTPQPSSPMDQMGKMRPQPYGGTNPYS<br>GGGGPPSGPQQGHGYPGQPYGSQTPQRYPMTMQGRAQSAMGGLSY<br>TQQIPPYGQQGPSGYGQQGQTPYYNQQSPHPGGGGPPYSQQPPSQT<br>PHAQPSYGGGPGSGPPGLGSSQPPYSQQPSQPPHQQSPAPYPSGGS<br>TTGGHPQSQPPYSQPQAQSPYGGGGPGGPAPSTLSQQAAYPGPGSG<br>GSGGTAYSQQRFPPPQ
ARID1A$^{\text{PLD YtoS}}$	Live cell imaging	MSNGGGGGGGAGSGGGPGAEPDLKNSNGNAGPRPALNNNLTEPPGG<br>GGGGSSDGVGAPPHSAAAALPPPASGFGQPSGRSPSAVAAAAAAVFH<br>QQHGGQQSPGLAALQSGGGGGLEPSAGPQQNSHDHGFPNHQSNSSS<br>PNRSASPPPAPASALSSPRGGTPGSGAAAAAGSKPPPSSSASASSSSS<br>SFAQQRFGAMGGGGPSAAGGGTPQPTATPTLNQLLTSPSSARGSQGS<br>PGGDSSGGPQDGGAGKGPADMASQCWGAAAAAAAAAAASGGAQQR<br>SHHAPMSPGSSGGGGQPLARTPQPSSPMDQMGKMRPQPSGGTNPSS<br>QQQGPPSGPQQGHGSPGQPSGSQTPQRSPMTMQGRAQSAMGGLSS<br>TQQIPPSGQQGPSGSGQQGQTPSSNQQSPHPQQQQPPSSQQPPSQT<br>PHAQPSSQQQPQSQPPQLQSSQPPSSQQPSQPPHQQSPAPSPSQQS<br>TTQQHPQSQPPSSQPQAQSPSQQQQPQQPAPSTLSQQAASPQPQSQ<br>QSQQTASSQQRFPPPQ

Table S4: List of protein constructs used for recombinant protein expression and purification and live cell imaging in this work. The mutation sites are highlighted in blue.

Plasmid	Tag	Source plasmid
BRG1^{PLD}	mEGFP	Synthesized by Genscript
ARID1A^{PLD}	mEGFP	Synthesized by Genscript
ARID1B^{PLD}	mEGFP	Synthesized by Genscript
SS18^{PLD}	mEGFP	Synthesized by Genscript
FUS^{PLD}	mEGFP	Synthesized by Genscript
FUS^{2XPLD}	mEGFP	Synthesized by Genscript
OptoFUS^{PLD}	CRY2-mCherry	Kind gift from Dr. Sreejith Nair
OptoFUS$^{PLD\text{-}NLS}$	CRY2-mCherry-NLS	Cloned by Genscript
BRG1^{PLD}	mCherry	Synthesized by Genscript
ARID1A^{PLD}	mCherry	Synthesized by Genscript
ARID1B^{PLD}	mCherry	Synthesized by Genscript
SS18^{PLD}	mCherry	Synthesized by Genscript
FUS^{PLD}	mCherry	Synthesized by Genscript
RNA Pol II CTD^{30}	mCherry	Synthesized by Genscript
mCherry2-C1		Addgene #54563, gift from Michael Davidson
SMARCC1^{PLD}	GFP	Synthesized by Genscript
EWSR1^{PLD}	GFP	Synthesized by Genscript
TAF15^{PLD}	GFP	Synthesized by Genscript
BRG1$^{PLD\ Aro+}$	GFP	Synthesized by Genscript
BRG1$^{PLD\ Aro++}$	GFP	Synthesized by Genscript
ARID1A$^{PLD\ YtoS}$	GFP	Synthesized by Genscript
ARID1A$^{PLD\ 30QtoG}$	GFP	Synthesized by Genscript
FOXG1$^{N\text{-}IDR}$	GFP	Synthesized by Genscript
FOXG1$^{N\text{-}IDR}$	mCherry	Synthesized by Genscript
FUS$^{2XPLD\ halfYtoS}$	GFP	Synthesized by Genscript
BRG1	GFP	Addgene #65391, gift from Kyle Miller
BRG1^{Folded}	GFP	Synthesized by Genscript
BRG1^{PLD} 2C	His-MBP-N10-Tev	Synthesized by Genscript
ARID1A^{PLD}	His-MBP-N10-Tev	Synthesized by Genscript
ARID1B^{PLD}	His-MBP-N10-Tev	Synthesized by Genscript
SS18^{PLD} 2C	His-MBP-N10-Tev	Synthesized by Genscript
FUS^{PLD} S86C	His-MBP-N10-Tev	Synthesized by Genscript
RNA Pol II CTD^{30} 2C	His-MBP-N10-Tev	Synthesized by Genscript
FOXG1$^{N\text{-}IDR}$ 2C	His-MBP-N10-Tev	Synthesized by Genscript
His-MBP-TEV-cys	His-MBP-N10-Tev	Synthesized by Genscript

Table S5: List of plasmids used in this work for protein expression in HEK293T cells.

Side chain chemistry	Amino acid	Color
Positively charged	Arg, Lys & His	Green
Negatively charged	Asp & Glu	Pink
Polar uncharged	Ser, Thr, Asn & Gln	Yellow
Hydrophobic	Ala, Val, Ile, leu, Met, Phe, Tyr & Trp	Blue
Others	Pro, Gly & Cys	Orange

Table S6: Color code for amino acids in Figs.1b, 2a, & 5b.

Supplementary Figures

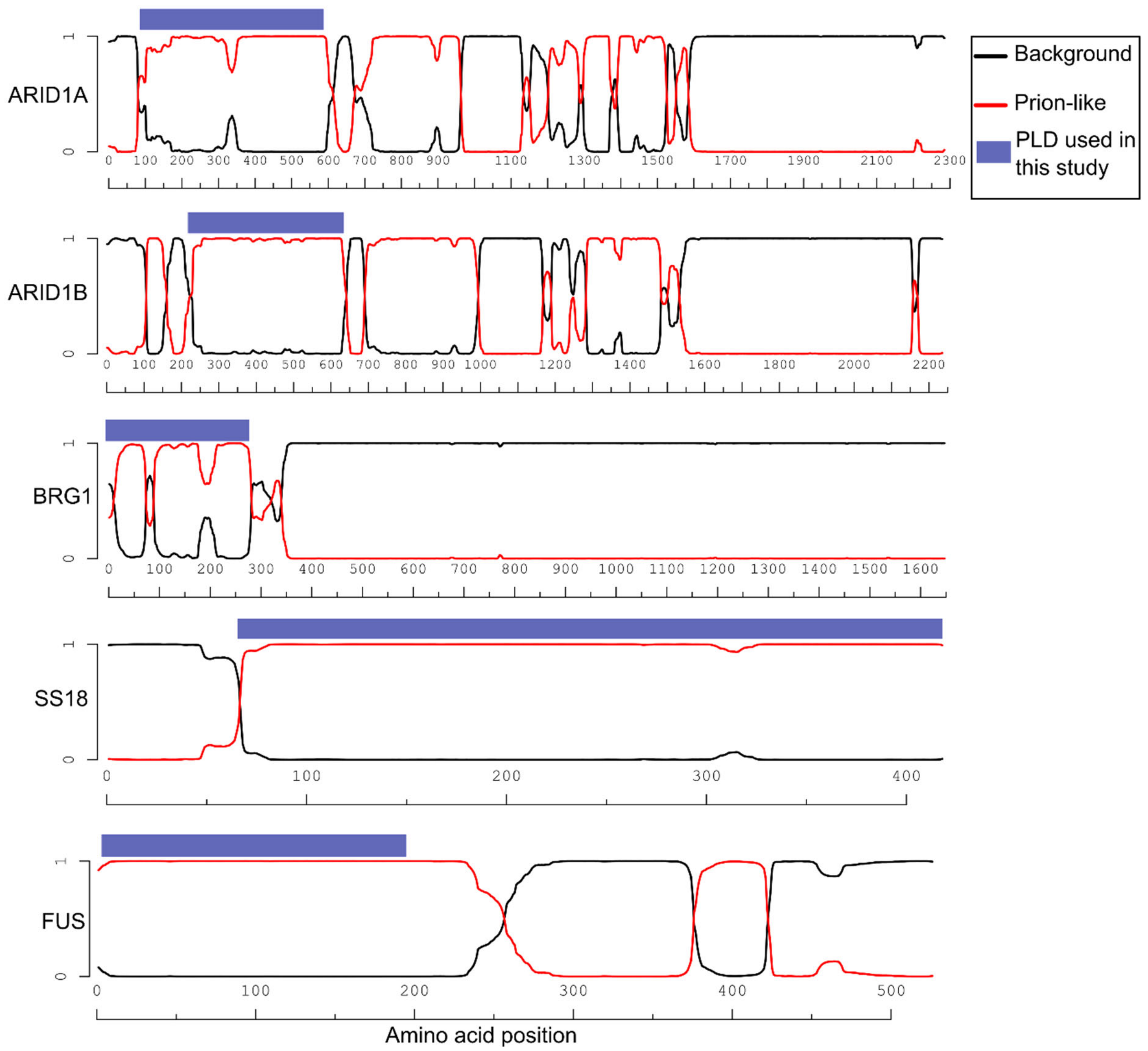

Figure S1: PLAAC analysis [2] showing regions with high prion-propensity for the four subunits of mSWI/SNF complex (ARID1A, ARID1B, BRG1, and SS18) and FUS. Domains corresponding to blue bars were used in this study.

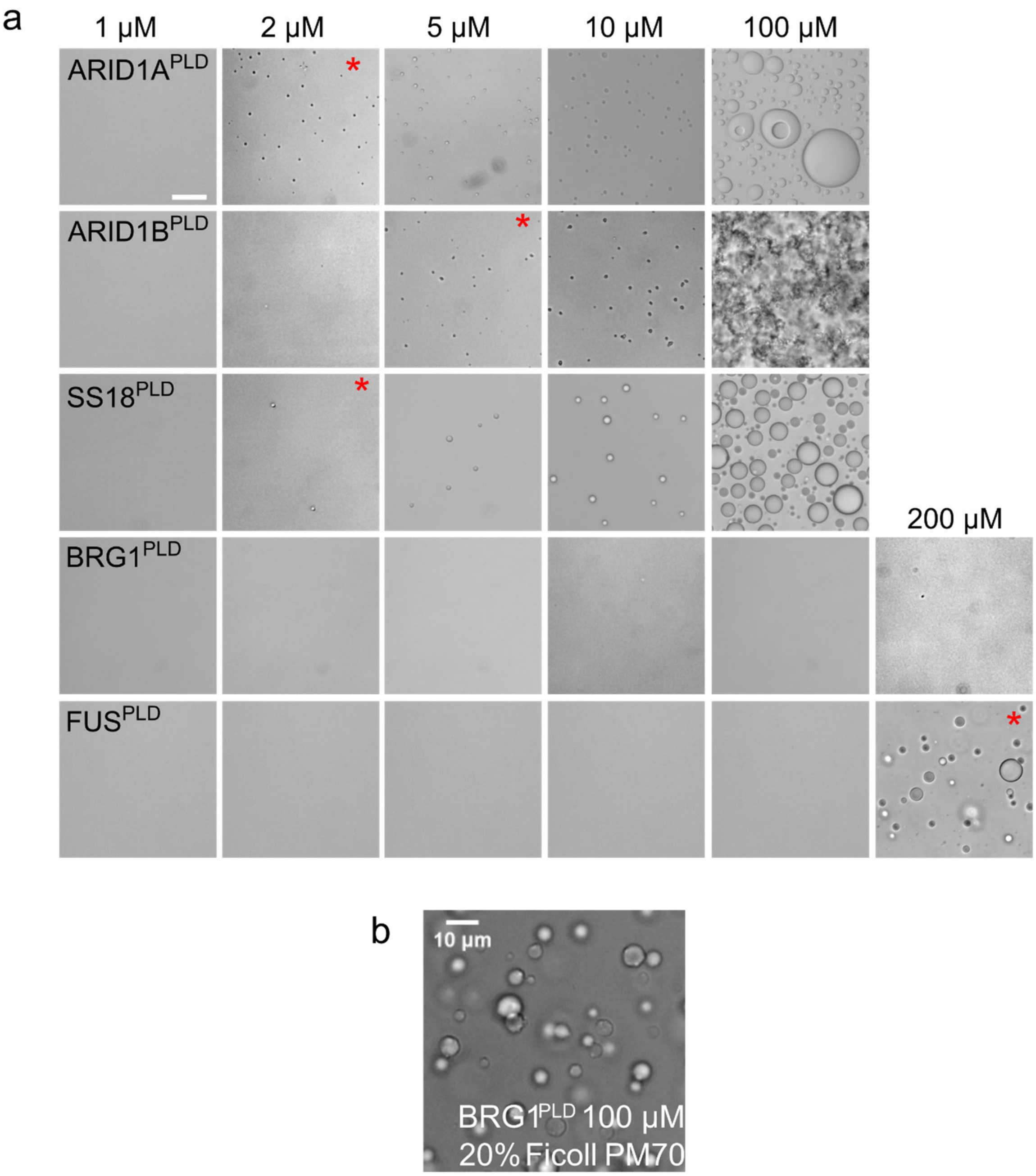

Figure S2: a) Differential interference contrast (DIC) microscopy images showing concentration titrations of recombinantly purified FUS^{PLD} and mSWI/SNF PLDs ($ARID1A^{PLD}$, $ARID1B^{PLD}$, $SS18^{PLD}$, and $BRG1^{PLD}$). The red asterisk indicates the concentration at which condensates were first observed. The scale bar is 10 μm. **b)** Phase separation of $BRG1^{PLD}$ was observed at 100 μM protein concentration with 20% Ficoll PM70.

a

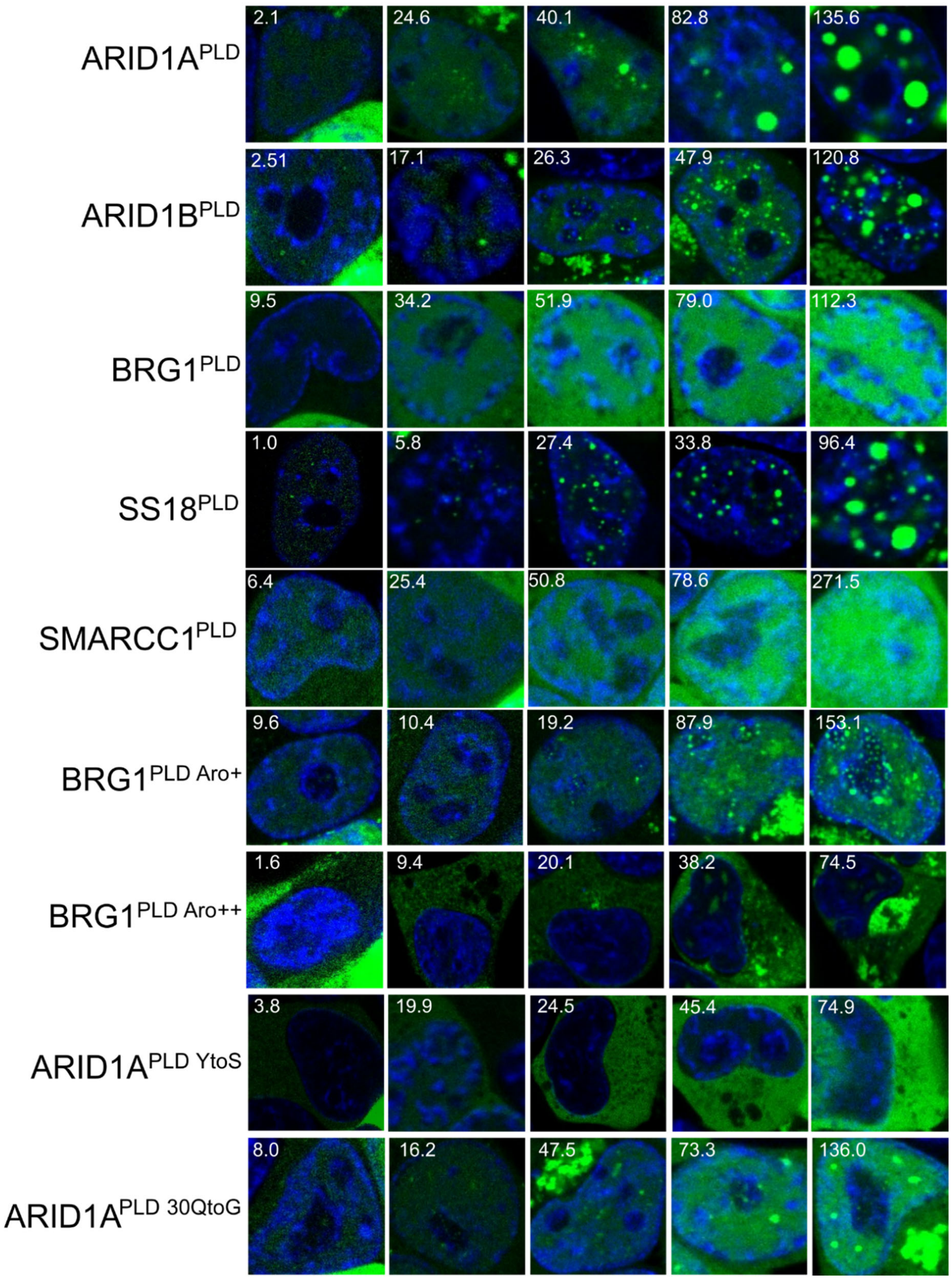
ARID1A^PLD
2.1
24.6
40.1
82.8
135.6
ARID1B^PLD
2.51
17.1
26.3
47.9
120.8
BRG1^PLD
9.5
34.2
51.9
79.0
112.3
SS18^PLD
1.0
5.8
27.4
33.8
96.4
SMARCC1^PLD
6.4
25.4
50.8
78.6
271.5
BRG1^PLD Aro+
9.6
10.4
19.2
87.9
153.1
BRG1^PLD Aro++
1.6
9.4
20.1
38.2
74.5
ARID1A^PLD YtoS
3.8
19.9
24.5
45.4
74.9
ARID1A^PLD 30QtoG
8.0
16.2
47.5
73.3
136.0

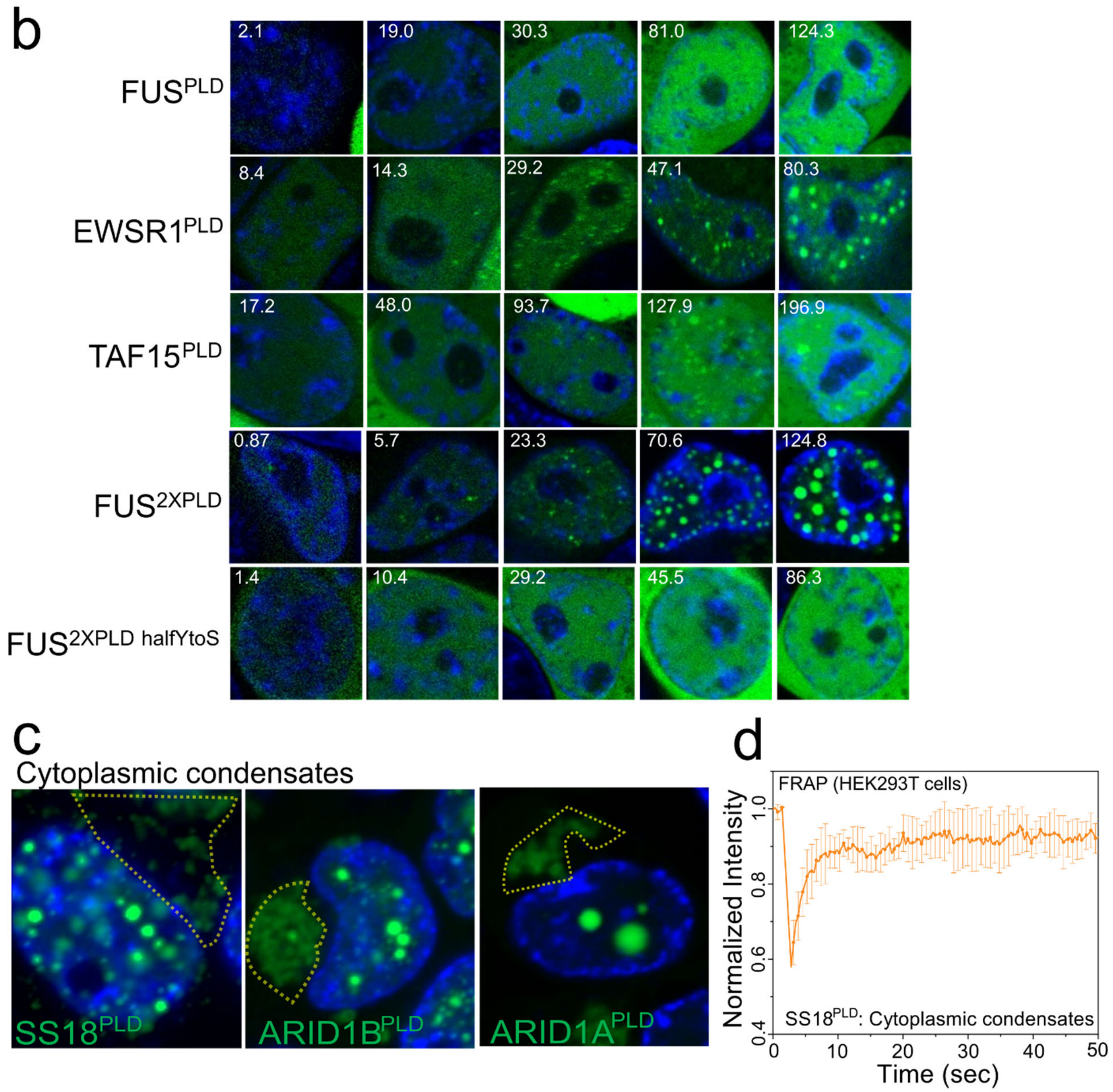

Figure S3: Fluorescence microscopy images of HEK293T cells expressing GFP-tagged PLDs and variants of **a)** mSWI/SNF subunits, and **b)** FET proteins at varying expression levels. The mean GFP intensity values are noted in each image. Hoechst was used to stain the cell nucleus, which is shown in blue. For BRG1$^{PLD\ Aro++}$ and ARID1A$^{PLD\ YtoS}$, mean cytoplasmic intensities are noted since their expression was predominantly cytoplasmic. For some mSWI/SNF subunit PLDs, higher expression level has been noted to result in the formation of irregular cytoplasmic condensates. **c)** Irregular-shaped cytoplasmic condensates of GFP-labeled PLDs in HEK293T cells are displayed within the yellow dashed lines. Hoechst was used to stain the cell nucleus, which is shown in blue. **d)** FRAP curve for cytoplasmic condensates of GFP-tagged SS18^{PLD} in HEK293T cells. The average intensity and standard deviation of the intensity profiles are shown as a function of time (n = 3 cells).

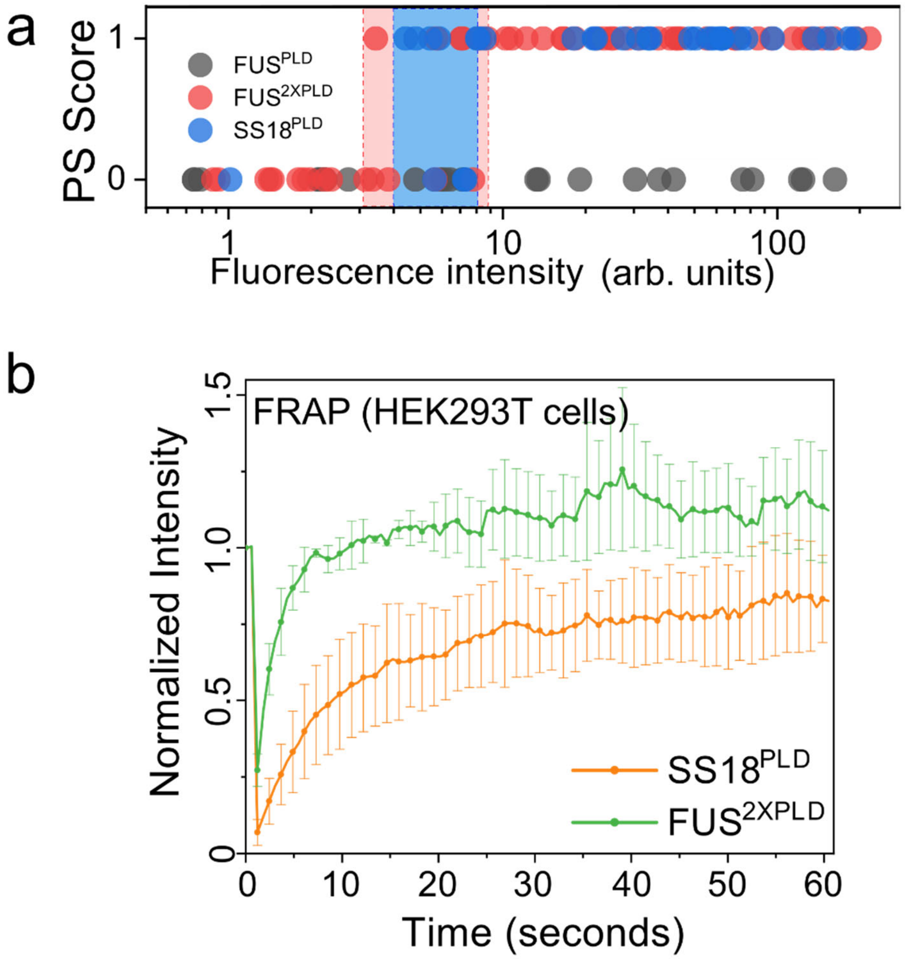

Figure S4: a) The phase separation capacity is quantified over various levels of nuclear protein concentration for FUS^{PLD}, FUS^{2XPLD}, and $SS18^{PLD}$ and presented as a state diagram. A phase separation (PS) score of '1' indicates the presence of nuclear condensates and a PS score of '0' represents diffused expression patterns. The shaded regions represent the transition concentrations. (FUS^{PLD} n = 25 cells, FUS^{2XPLD} n = 51 cells, $SS18^{PLD}$ n = 32 cells, from two biological replicates). **b)** FRAP curve for condensates of GFP-tagged FUS^{2XPLD} and $SS18^{PLD}$ in HEK293T cells. The average intensity and standard deviation of the intensity profiles are shown over time (n = 3 cells).

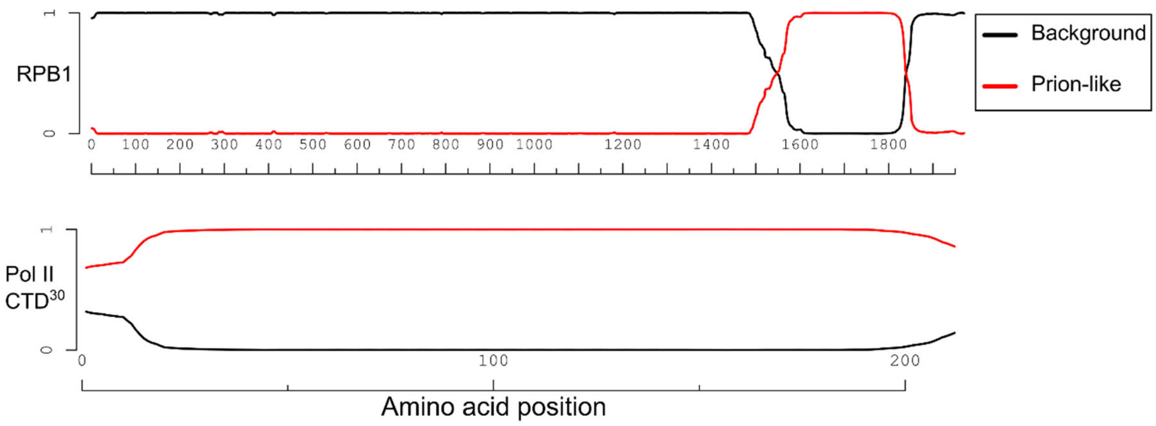

Figure S5: PLAAC[2] analysis showing regions with high prion-propensity for the human RNA Polymerase II subunit RPB1 (*Top panel*) and the first 30 repeats of the heptad (Pol II CTD[30]) used in this study (*bottom panel*).

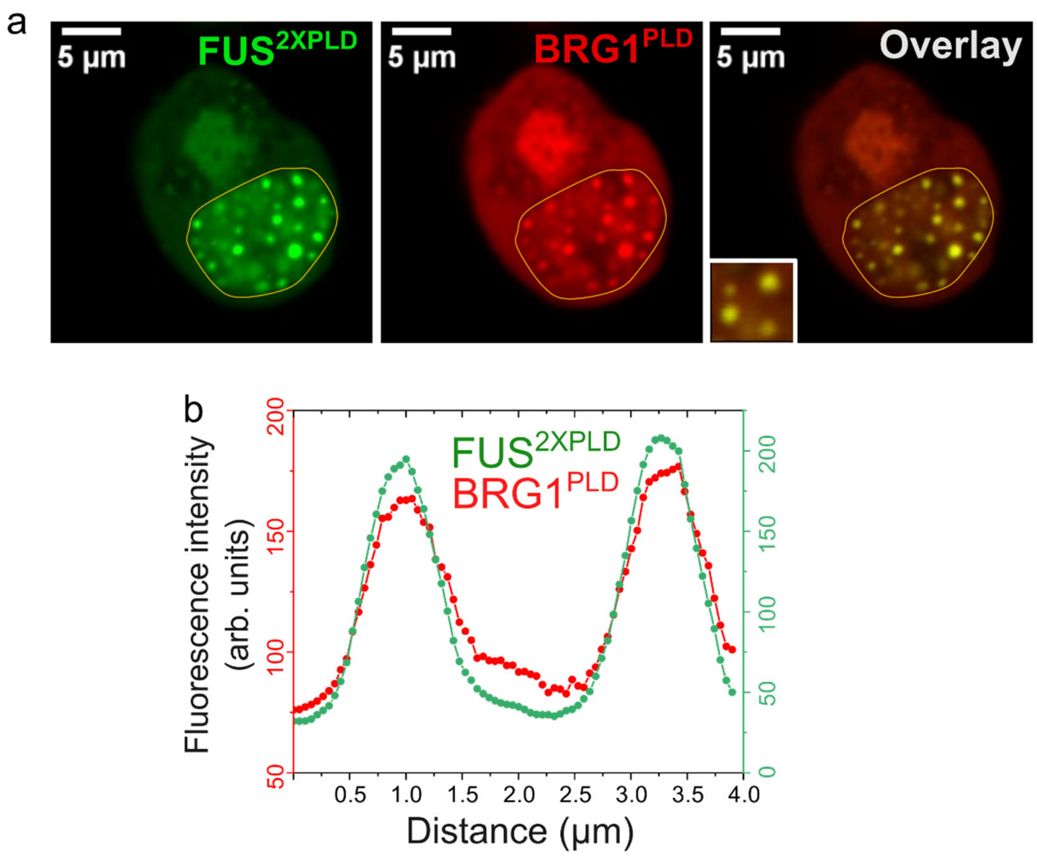

Figure S6: a) HEK293T cells co-expressing GFP-FUS^{2XPLD} and mCherry-BRG1^{PLD}. **b)** The degree of colocalization is displayed as intensity profiles for condensates shown in the inset image. Green represents the intensity profile of GFP-FUS^{2XPLD} and red represents the intensity profile for mCherry-BRG1^{PLD}.

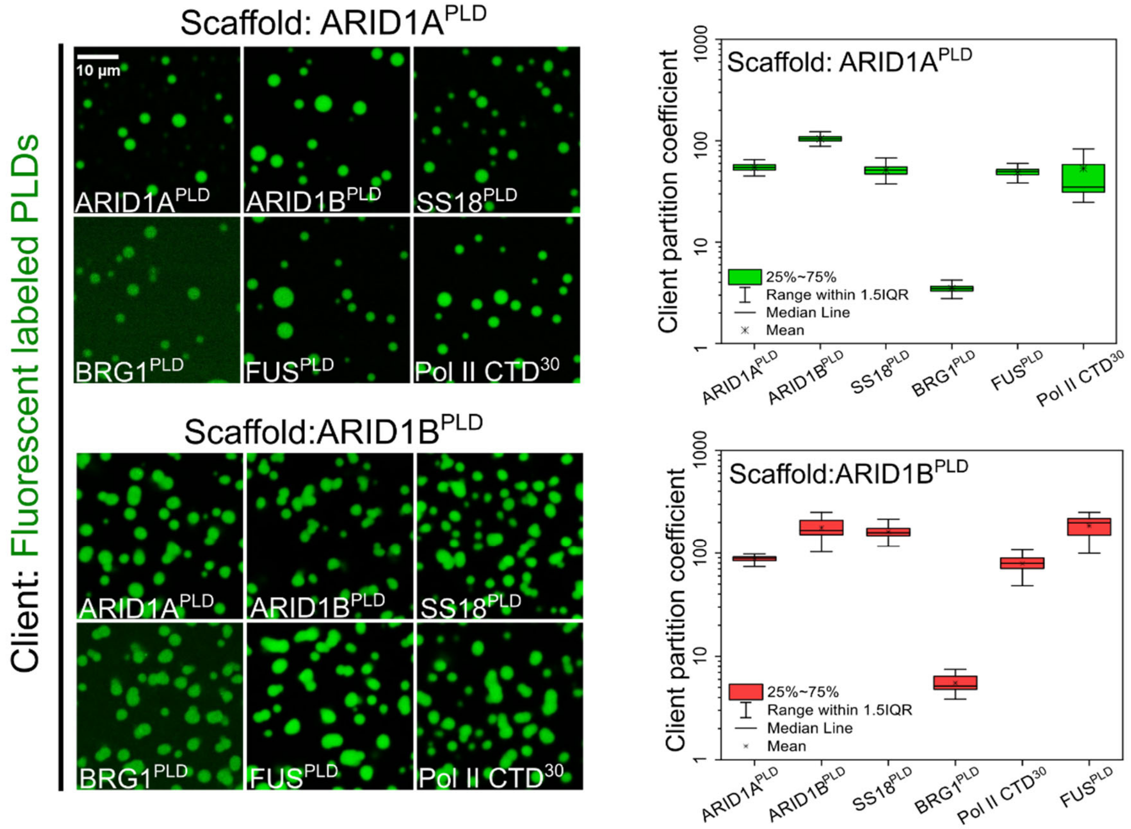

Figure S7: Partitioning of AlexaFluor488 labeled client PLDs (ARID1A^{PLD}, ARID1B^{PLD}, SS18^{PLD}, BRG1^{PLD}, RNA Polymerase II CTD^{30}, and FUS^{PLD}) within condensates of ARID1A^{PLD} and ARID1B^{PLD}. Enrichment (partition coefficient) is calculated as shown in Fig. 3 in the main text and displayed as a box-and-whisker plot. (ARID1A^{PLD} condensates: ARID1A^{PLD} n = 147 condensates, ARID1B^{PLD} n = 203 condensates, SS18^{PLD} n = 344 condensates, BRG1^{PLD} n = 272 condensates, RNA Polymerase II CTD^{30} n = 180 condensates, and FUS^{PLD} n = 278 condensates; ARID1B^{PLD} condensates: ARID1A^{PLD} n = 561 condensates, ARID1B^{PLD} n = 552 condensates, SS18^{PLD} n = 690 condensates, BRG1^{PLD} n = 353 condensates, RNA Polymerase II CTD^{30} n = 618 condensates, and FUS^{PLD} n = 568 condensates).

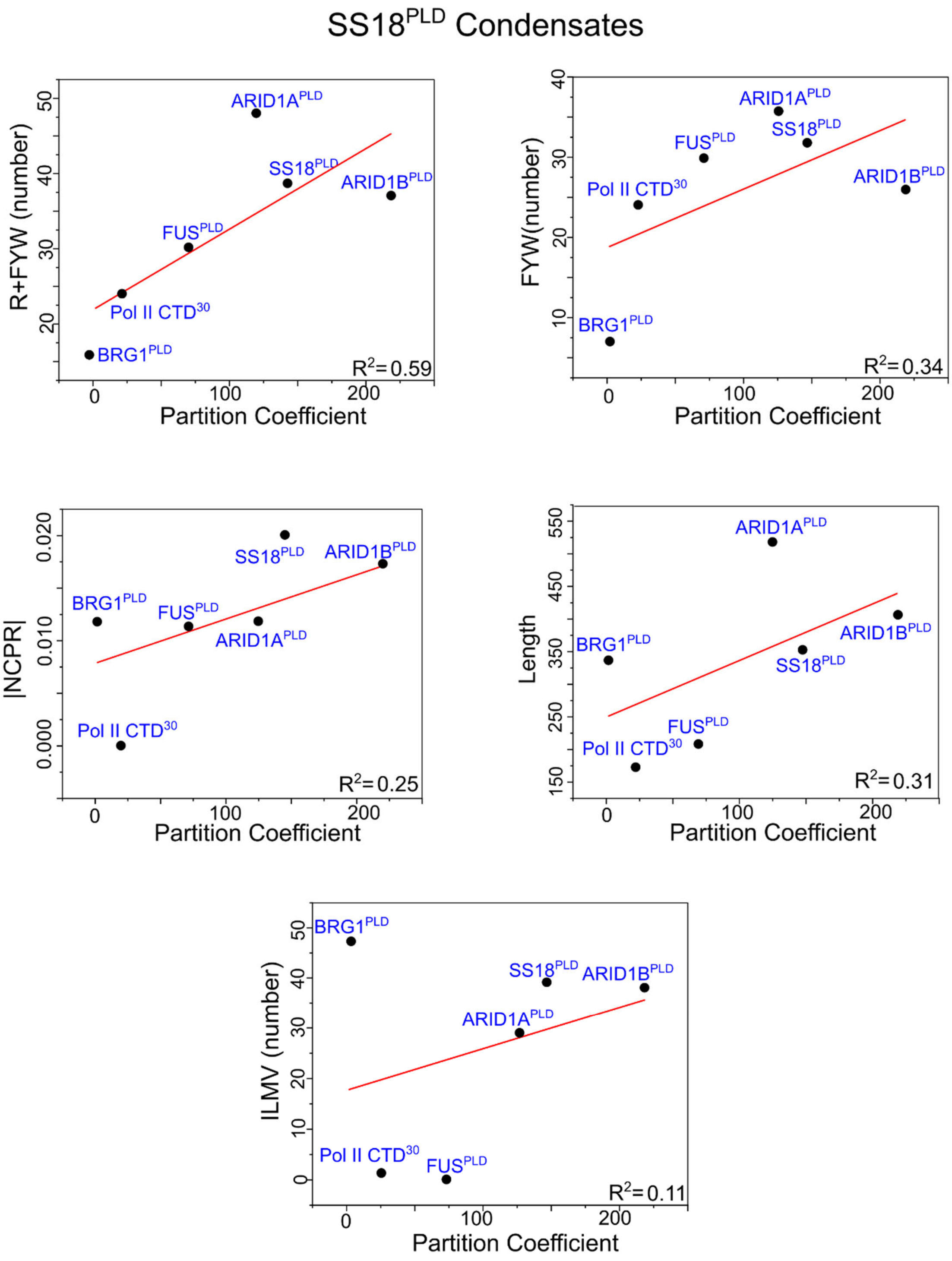

Figure S8: Linear regression analysis of in vitro partition coefficients in condensates formed by 50 μM SS18^{PLD} against various sequence features of the client PLDs (ARID1A^{PLD}, SS18^{PLD}, ARID1B^{PLD}, FUS^{PLD}, Pol II CTD^{30}, and BRG1^{PLD}). Sequence features included in the analysis are FYW: phenylalanine, tyrosine, and tryptophan residues representing the aromatic side chains; R: arginine; NCPR: net charge per residue; Chain length; ILMV: isoleucine, leucine, methionine, and valine representing the hydrophobic residues.

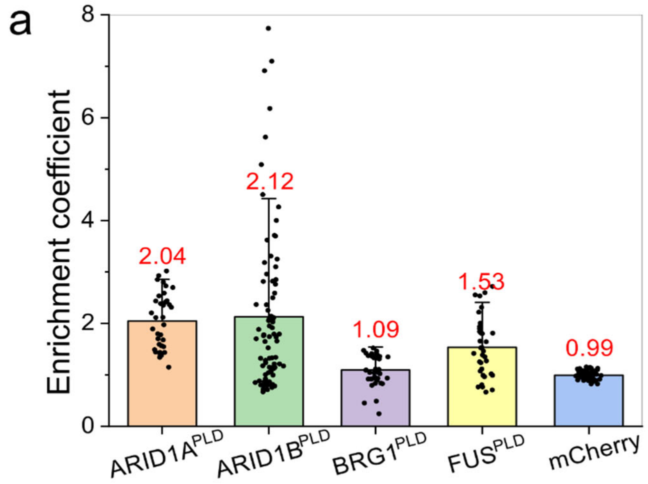

p-value	**ARID1B^{PLD}**	**BRG1^{PLD}**	**FUS^{PLD}**	**mCherry**
**ARID1A^{PLD}**	7.49E-01	3.49E-15	1.56E-04	2.62E-29
**ARID1B^{PLD}**		5.87E-05	2.48E-02	1.12E-08
**BRG1^{PLD}**			9.80E-05	5.58E-04
**FUS^{PLD}**				2.00E-11

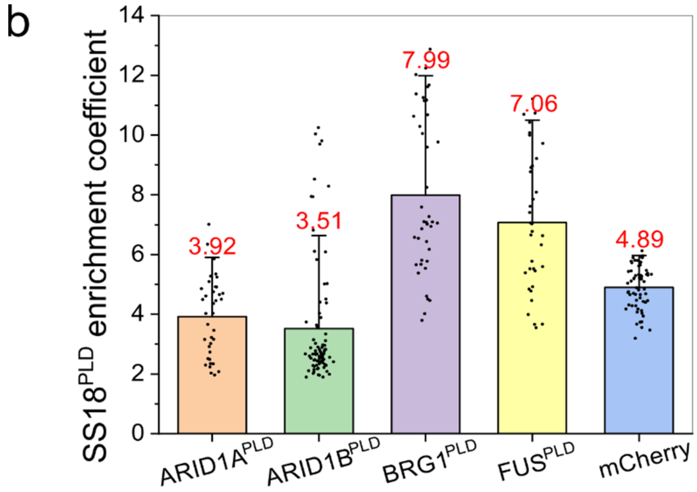

Figure S9: a) Enrichment coefficients of mCherry-tagged PLDs (ARID1A^{PLD}, ARID1B^{PLD}, BRG1^{PLD}, FUS^{PLD}, and mCherry alone) within condensates formed by GFP-SS18^{PLD} in HEK293T cells. Enrichment is calculated as the ratio of mean intensities from the dense phase and the dilute phase. The average enrichment coefficient score is shown in red for each PLD and the mCherry control. (ARID1A^{PLD} n = 37 condensates, ARID1B^{PLD} n = 84 condensates, BRG1^{PLD} n = 39 condensates, FUS^{PLD} n = 35 condensates, and mCherry n = 65 condensates from two independent replicates). Student's two-tail t-test was used to calculate significance for each of the constructs and the p-values are tabulated. **b)** Self-partitioning of GFP-SS18^{PLD} within condensates formed when co-expressed with different mCherry-tagged client PLDs as indicated.

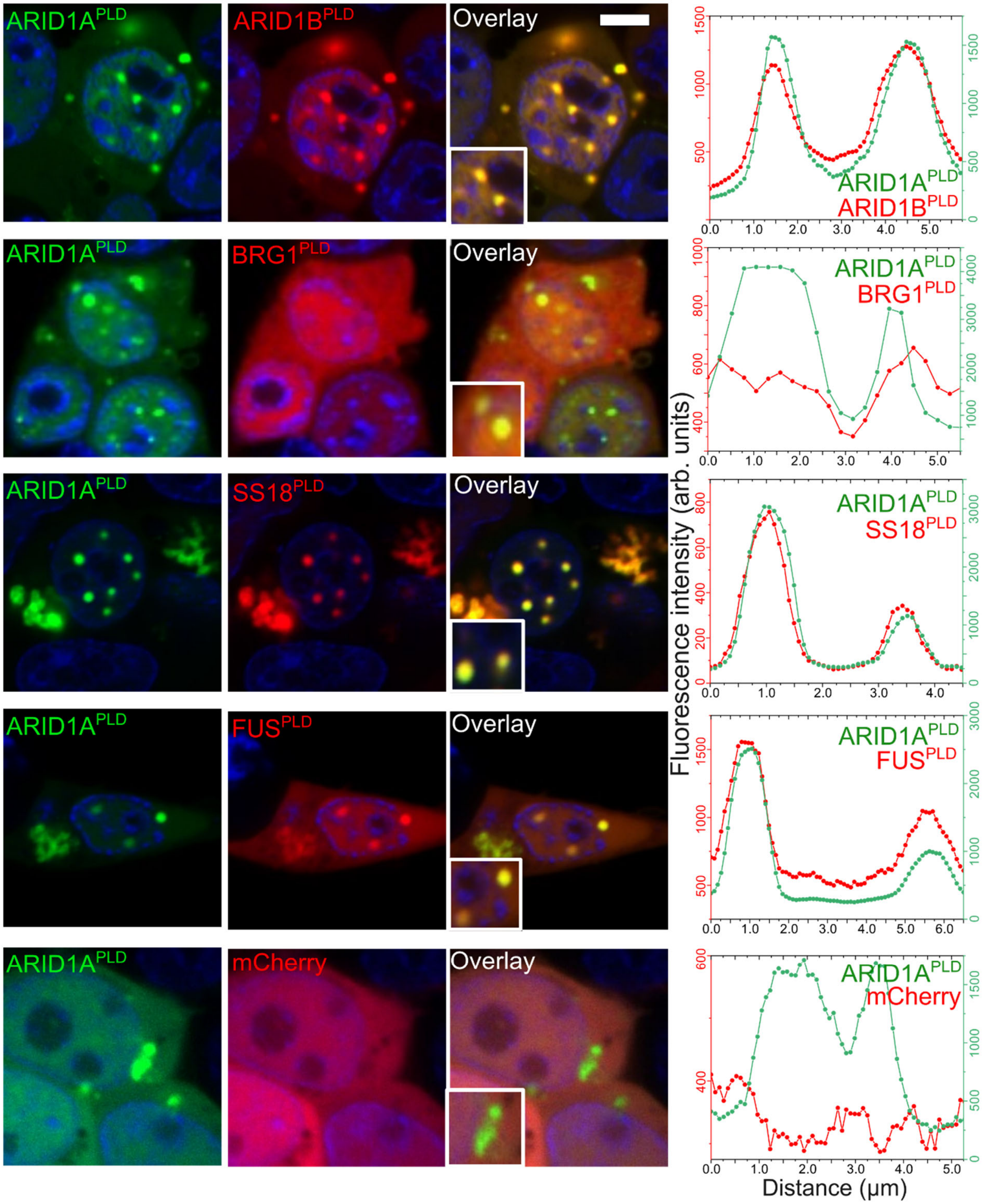

Figure S10: HEK293T cells co-expressing GFP-ARID1A^{PLD} and either one of the mCherry-tagged PLDs (ARID1B^{PLD}, BRG1^{PLD} , SS18^{PLD}, and FUS^{PLD}) or mCherry alone. The degree of co-localization is displayed as intensity profiles for condensates shown in the inset images. Green represents the intensity profile of GFP-ARID1A^{PLD} and red represents the intensity profile for mCherry-PLD. The scale bar is 10 μm.

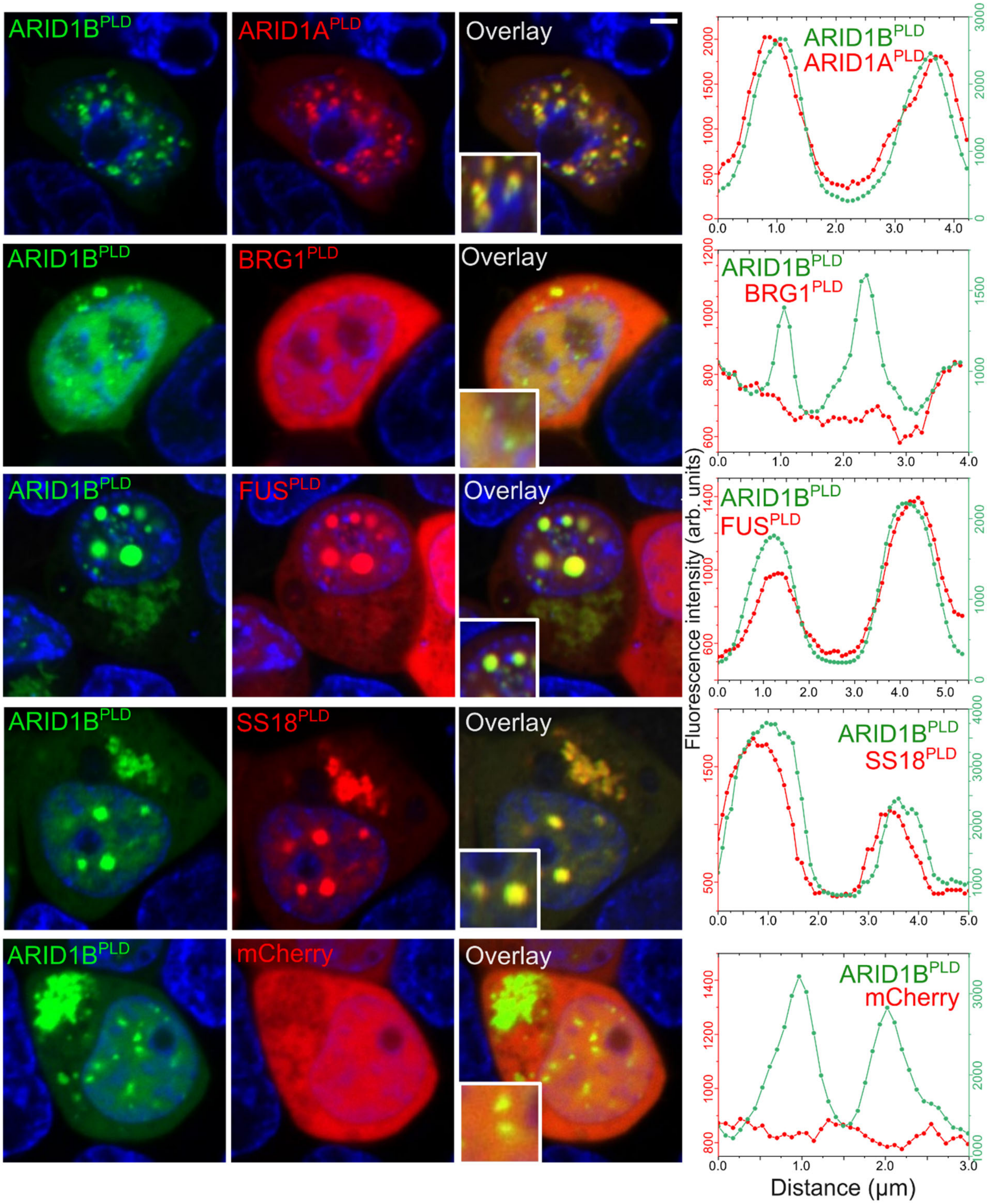

Figure S11: HEK293T cells co-expressing GFP-ARID1B^{PLD} and either one of the mCherry-tagged PLDs (ARID1A^{PLD}, BRG1^{PLD} , SS18^{PLD}, and FUS^{PLD}) or mCherry alone. The degree of colocalization is displayed as intensity profiles for condensates shown in the inset images. Green represents the intensity profile of GFP-ARID1B^{PLD} and red represents the intensity profile for mCherry-PLD. The scale bar is 5 μm.

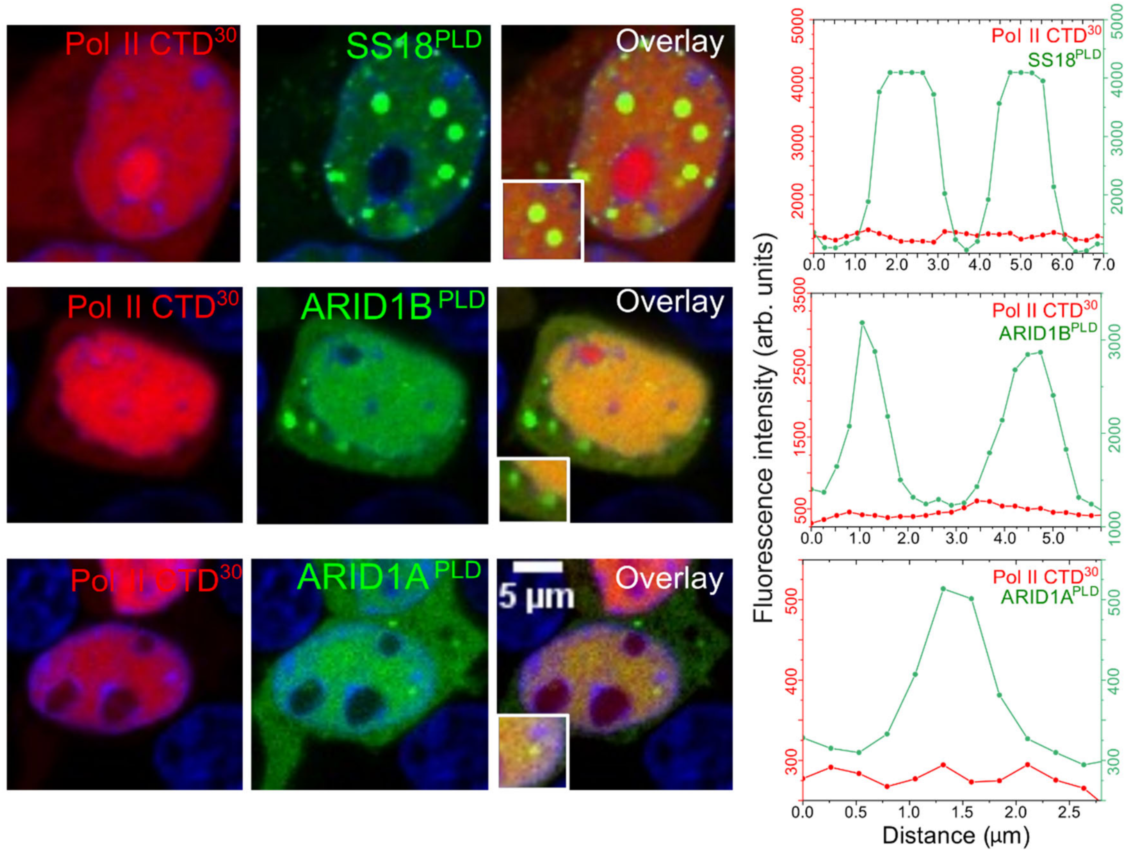

Figure S12: HEK293T cells co-expressing mCherry-RNA Pol II CTD^{30} and either one of the GFP tagged PLDs ($ARID1A^{PLD}$, $ARID1B^{PLD}$, $SS18^{PLD}$). The intensity profile is shown for condensates in the inset images. The degree of colocalization is displayed as intensity profiles for condensates present in the inset images. Green represents the intensity profile of GFP-PLDs and red represents the intensity profile for mCherry-Pol II CTD^{30}.

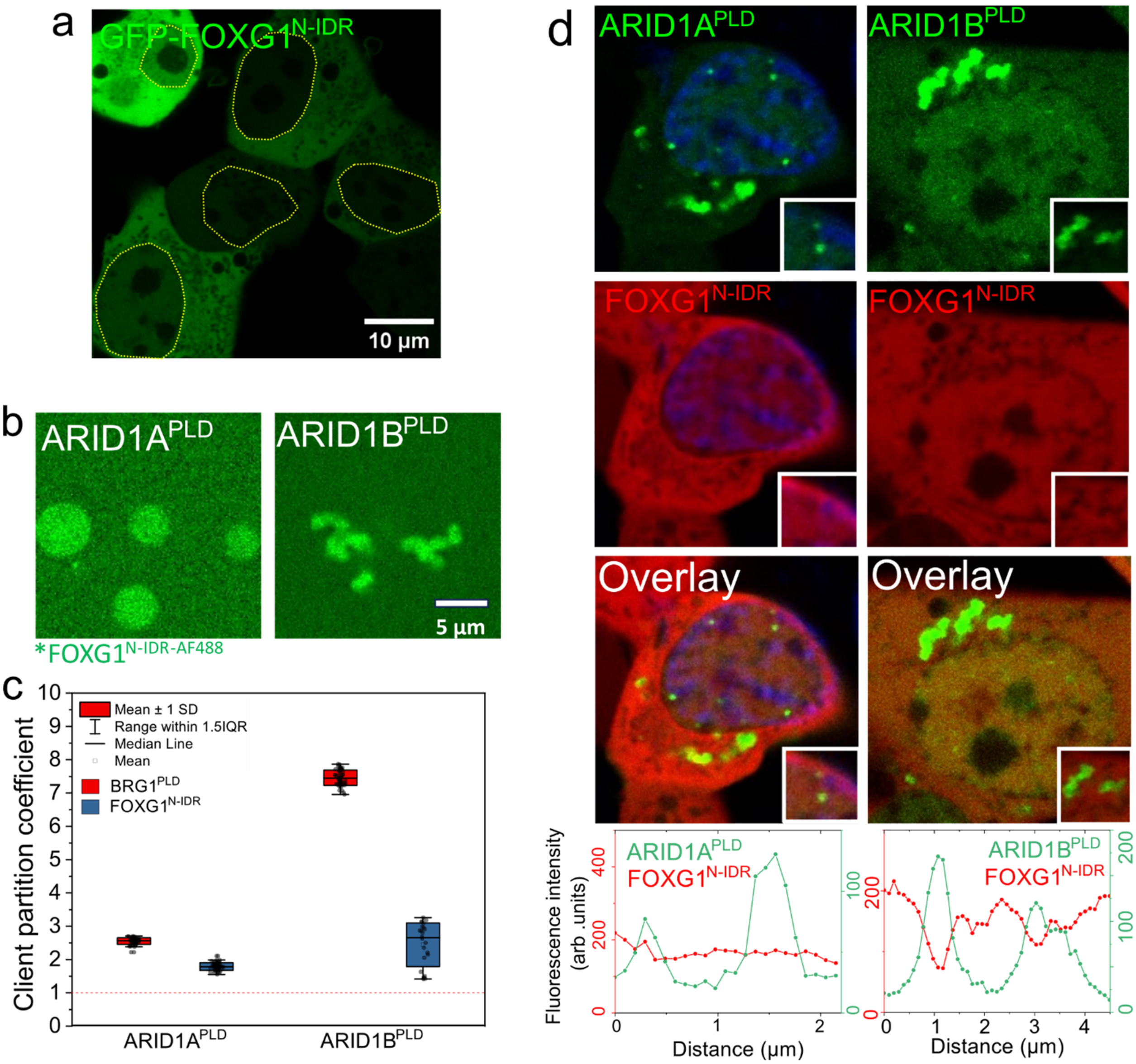

Figure S13: a) Fluorescence microscopy image of HEK293T cells expressing GFP-tagged FOXG1^{N-IDR}. Yellow dashed lines indicate the nuclear periphery. **b)** Partitioning of AlexaFluor488 labeled FOXG1^{N-IDR} within condensates formed by ARID1A^{PLD} and ARID1B^{PLD} (50 µM), respectively. **c)** Enrichment is calculated as partition coefficient and displayed as a box-and-whisker plot for both FOXG1^{N-IDR} and BRG1^{PLD} (data reported in Fig. S7) within these condensates (ARID1A^{PLD} condensates: FOXG1^{N-IDR} n = 104 condensates, BRG1^{PLD} n = 272 condensates; ARID1B^{PLD} condensates: FOXG1^{N-IDR} n = 24 condensates, BRG1^{PLD} n = 353 condensates). **d)** HEK293T cells co-expressing GFP-ARID1A^{PLD} or GFP-ARID1B^{PLD} and mCherry-tagged FOXG1^{N-IDR}.The degree of colocalization is displayed as intensity profiles for condensates shown in the inset images. Green represents the intensity profile of the GFP-tagged construct and red represents the profile for mCherry-tagged construct.

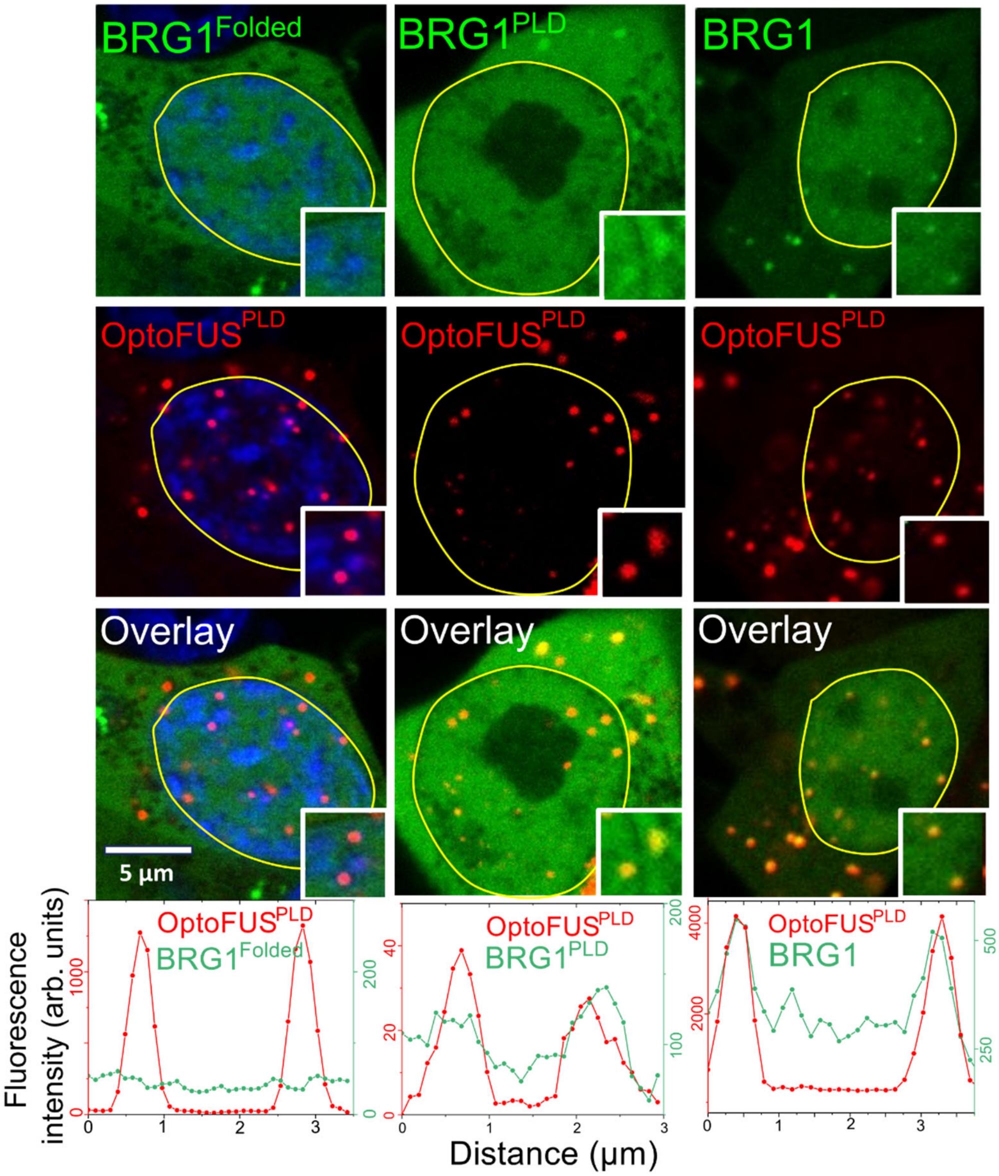

Figure S14: HEK293T cells co-expressing BRG1^{PLD}, BRG1^{Folded} or full-length BRG1 and mCherry-tagged OptoFUS^{PLD} construct. The degree of colocalization is displayed as intensity profiles for condensates shown in the inset images. Green represents the intensity profile of the GFP-tagged construct and red represents the profile for mCherry-tagged construct. Yellow lines indicate the nuclear periphery.

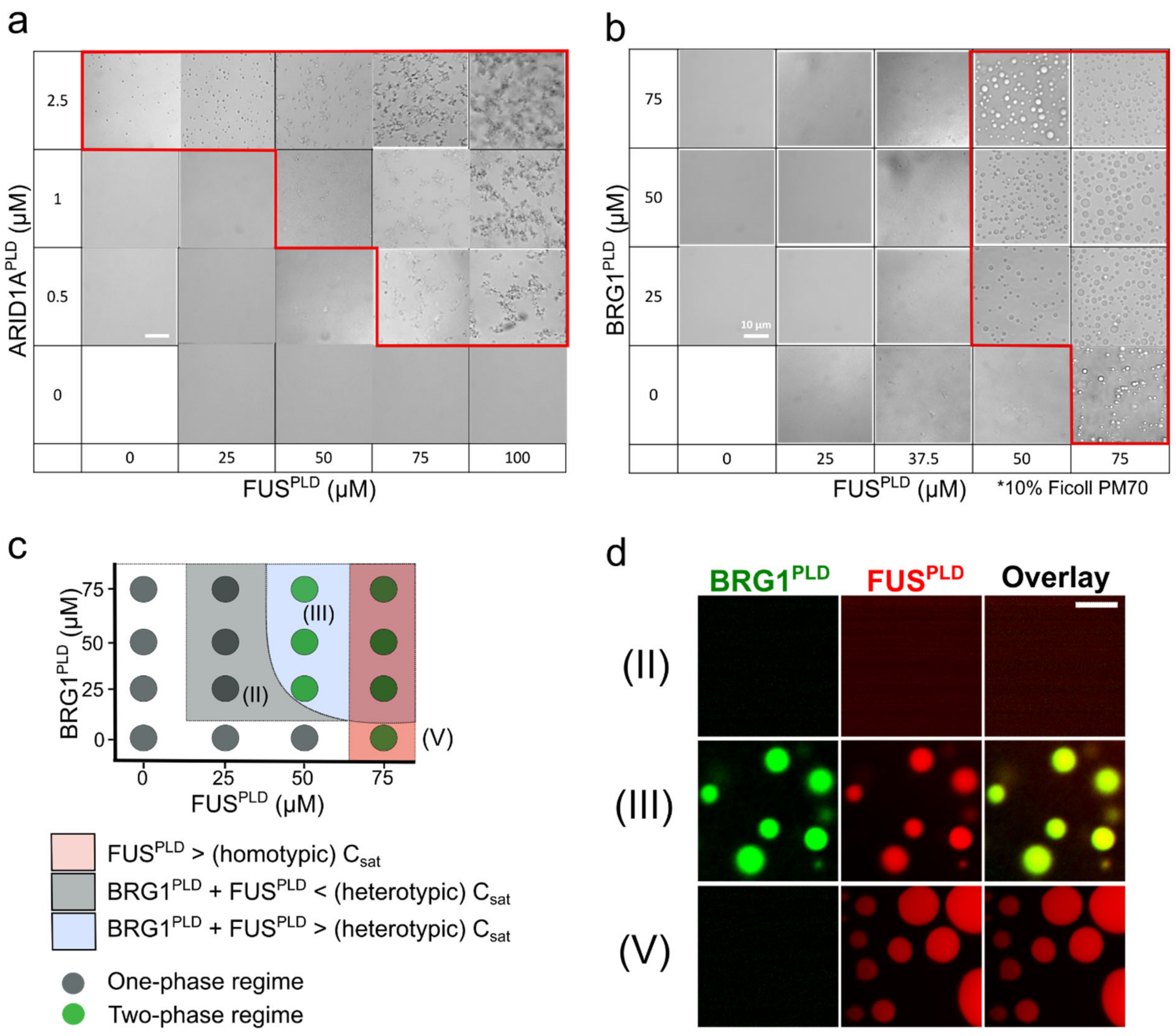

Figure S15: DIC images of the heterotypic PLD mixtures containing FUS^{PLD} and either **a)** ARID1A^{PLD} or **b)** BRG1^{PLD} at indicated concentrations. The images within the red-boxed region represent the two-phase regime. The FUS^{PLD} and BRG1^{PLD} ternary mixture contained 10% Ficoll PM70 in the buffer. **c)** Co-phase diagram of FUS^{PLD} and BRG1^{PLD} showing a decrease in saturation concentration of heterotypic PLD mixtures (regime III). The green circles indicate a two-phase regime, and the grey circles indicate a single-phase regime. The legend describes the shaded regions. **d)** Fluorescence microscopy images of samples from the indicated regions on the phase diagram in (**c**). These images show the formation of monophasic co-condensates of FUS^{PLD} and BRG1^{PLD}. BRG1^{PLD} is labeled with AlexaFluor488 and FUS^{PLD} is labeled with AlexaFluor594. The scale bar is 5 microns.

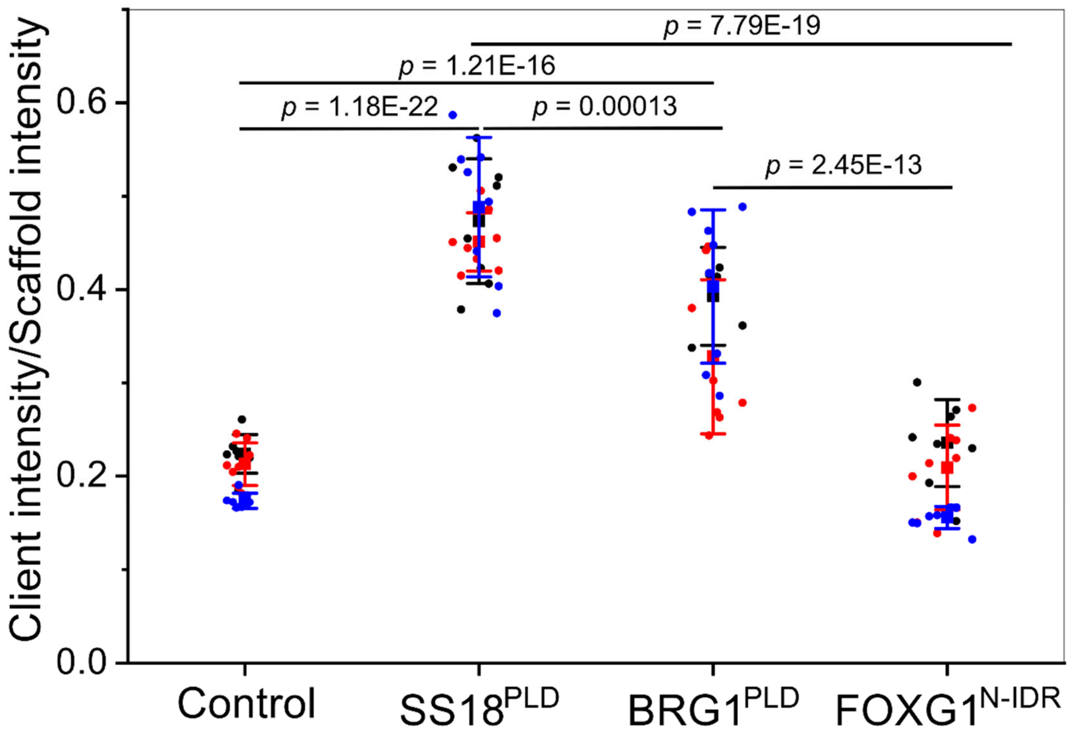

Figure S16: Statistical analysis for the bead halo assay data shown in Fig. 7b-c in the main text. Three trials were combined (with points for each of the trials shown in different colors) to calculate the significance using Student's two-tail t-test. Here the control refers to the AlexaFluor488-labeled His_6-MBP containing a short linker peptide (GGGCGGG) without any PLDs.

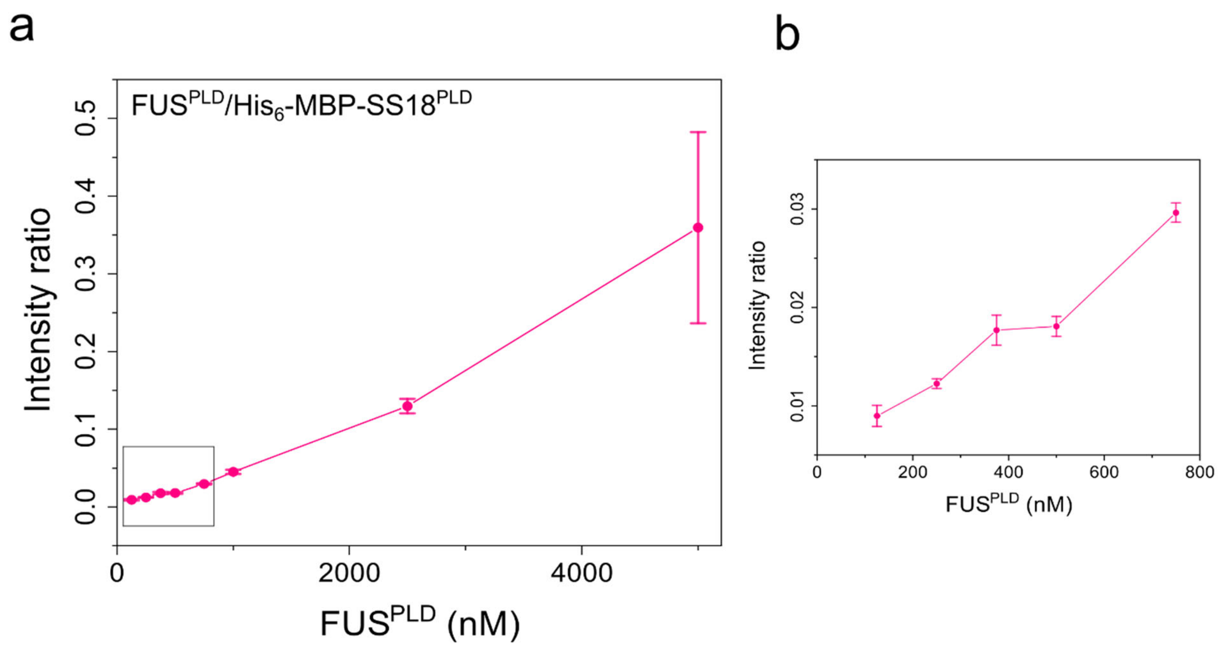

Figure S17: a) 250 nM of AlexaFluor488 labeled His$_6$-MBP-SS18^{PLD} was attached to Ni-NTA beads. AlexaFluor594 labeled FUS^{PLD} was titrated from 125 nM to 5 µM. Binding was quantified using the ratio of the fluorescence intensity (fluorescence signal from FUS^{PLD}/fluorescence signal from SS18^{PLD}) on the surface of the beads and plotted as a line plot with mean and standard deviation (n = 5 beads / data point). **b)** The region within the square box in (a) is enlarged and displayed here.

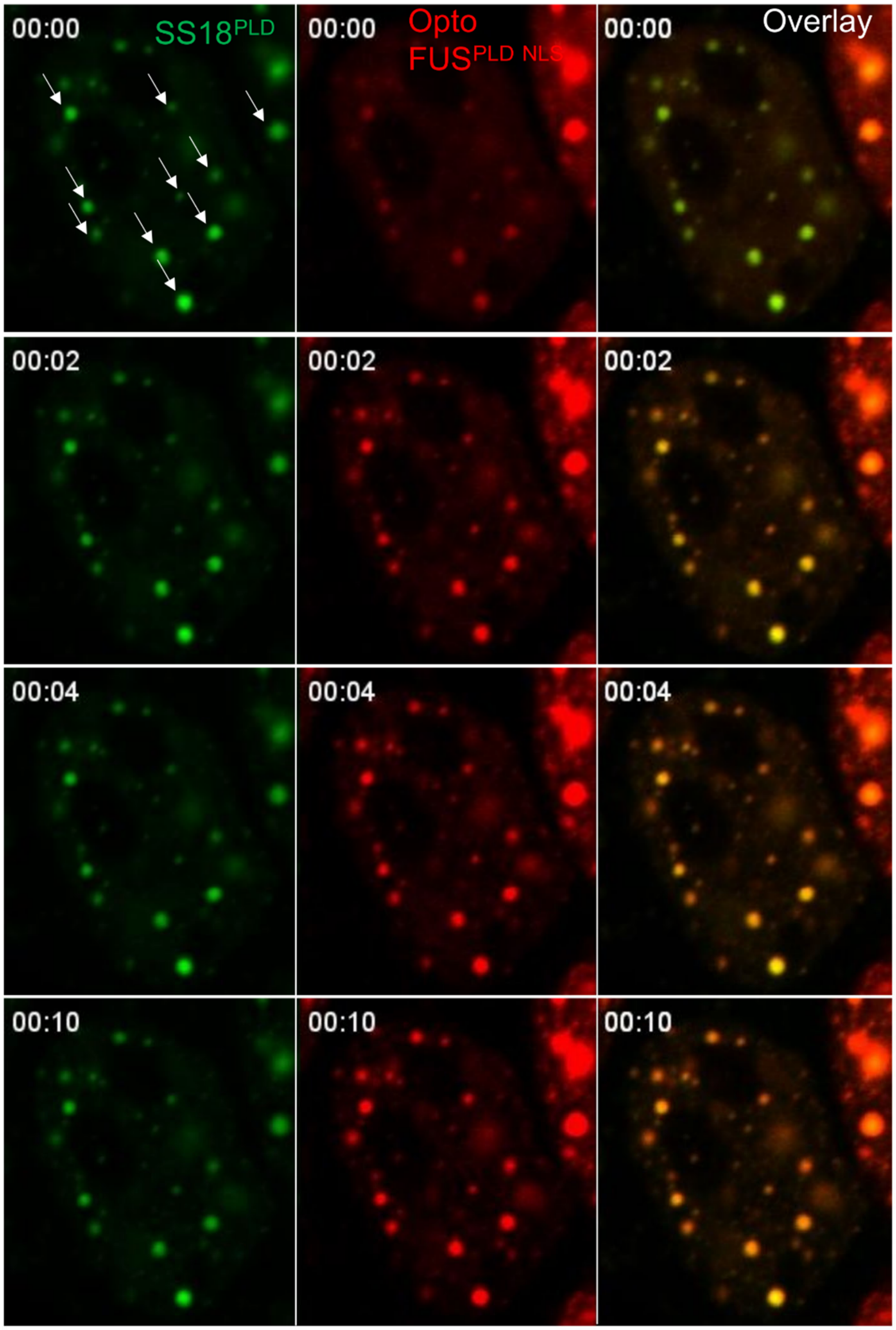

Figure S18. HEK293T cells co-expressing OptoFUS$^{PLD\text{-}NLS}$ (Cry2-mCherry-FUS$^{PLD\text{-}NLS}$) and GFP-SS18^{PLD}. Pre-existing GFP-SS18^{PLD} condensates, which are indicated by white arrows, serve as seeds to nucleate OptoFUS$^{PLD\text{-}NLS}$ condensates upon blue light activation. The corresponding video is shown as supplementary movie 3.

a

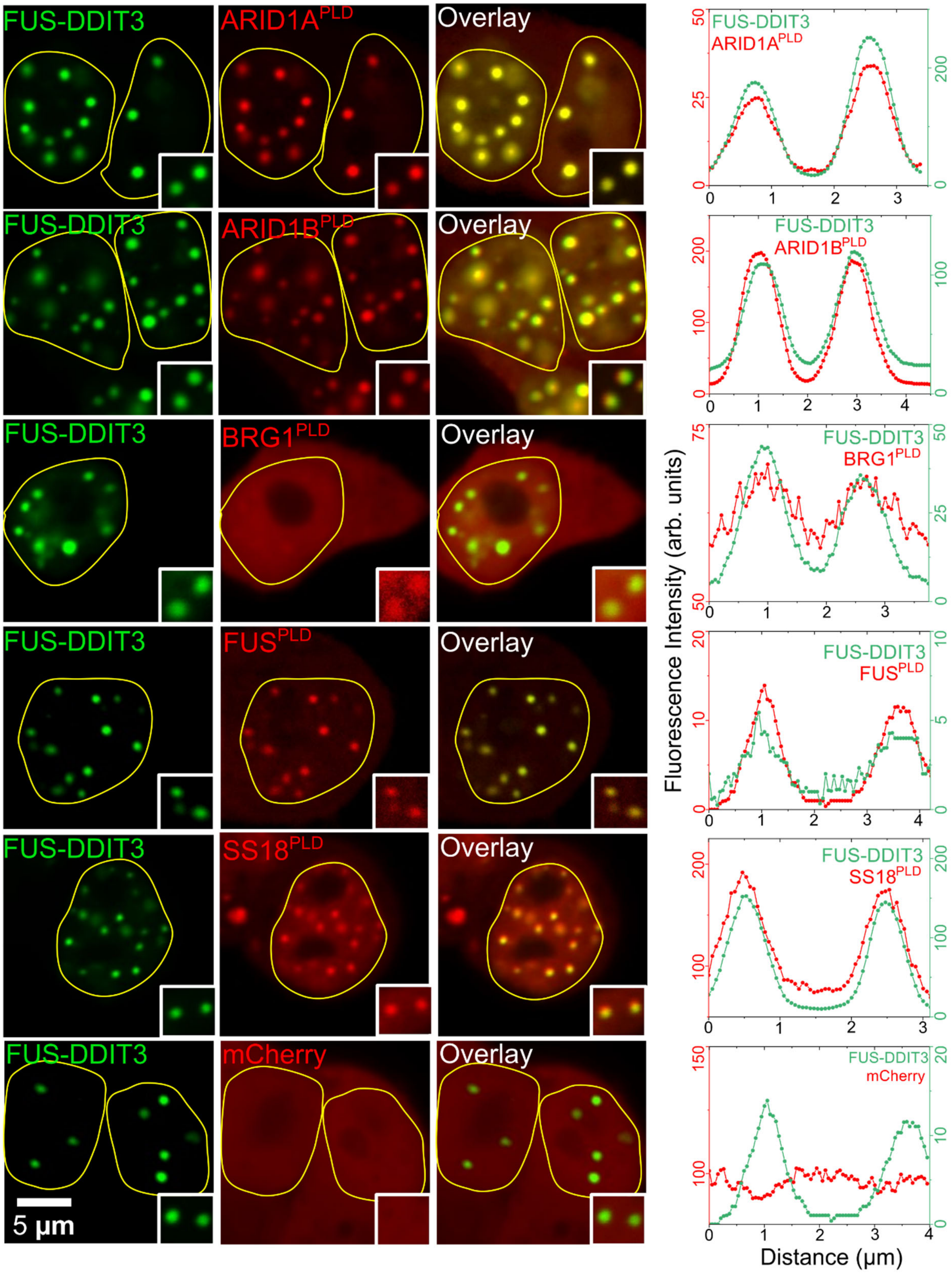

FUS-DDIT3
ARID1A^PLD
Overlay
ARID1B^PLD
BRG1^PLD
FUS^PLD
SS18^PLD
mCherry
5 µm
Fluorescence Intensity (arb. units)
Distance (µm)

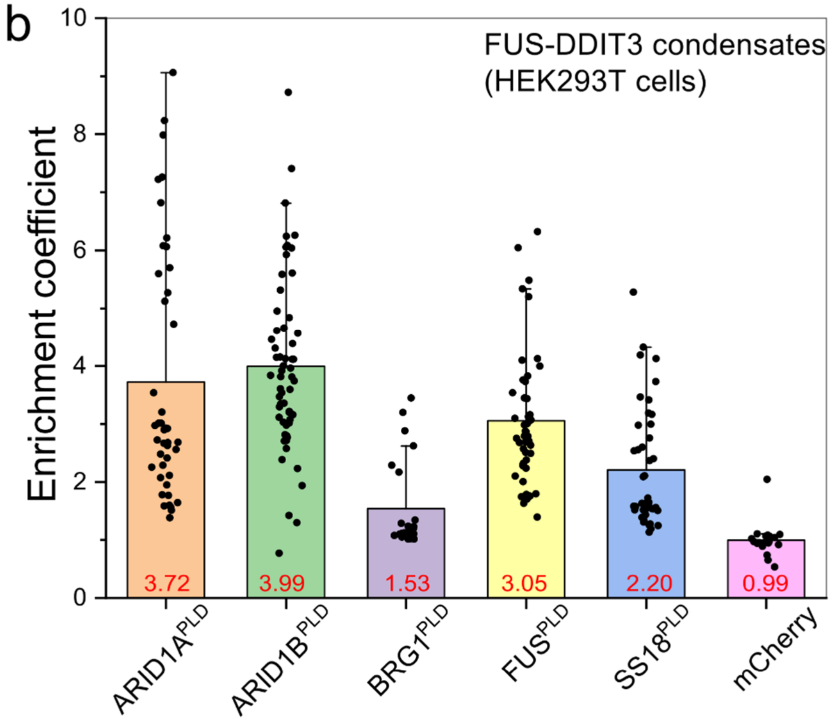

p-value	**ARID1B^{PLD}**	**BRG1^{PLD}**	**FUS^{PLD}**	**SS18^{PLD}**	**mCherry**
**ARID1A^{PLD}**	4.46E-01	9.72E-06	6.85E-02	6.03E-05	1.56E-07
**ARID1B^{PLD}**		2.69E-11	5.62E-04	4.35E-10	1.47E-14
**BRG1^{PLD}**			2.11E-07	6.77E-03	3.07E-03
**FUS^{PLD}**				4.01E-04	1.23E-11
**SS18^{PLD}**					9.17E-07

Figure S19: a) HEK293T cells co-expressing GFP-FUS-DDIT3 and either one of the mCherry-tagged PLDs (ARID1A^{PLD}, ARID1B^{PLD}, BRG1^{PLD}, FUS^{PLD}, and SS18^{PLD}) or mCherry alone. The degree of colocalization is displayed as intensity profiles for condensates shown in the inset images. Green represents the intensity profile of GFP-FUS-DDIT3 and red represents the intensity profile for mCherry-PLD. The nuclear periphery is highlighted in yellow. **b)** Enrichment is calculated as the ratio of mean intensities from the dense phase and the dilute phase. The average enrichment coefficient score is shown in red for each client. (ARID1A^{PLD} n = 43, ARID1B^{PLD} n = 61, BRG1^{PLD} n = 24, FUS^{PLD} n = 46, SS18^{PLD} n = 44, and mCherry n = 22). Student's two-tail t-test was used to calculate significance for each of the constructs and the p-values are tabulated.

References

1. Holehouse, A. S., Das, R. K., Ahad, J. N., Richardson, M. O. G. & Pappu, R. V. CIDER: Resources to Analyze Sequence-Ensemble Relationships of Intrinsically Disordered Proteins. *Biophys. J.* **112**, 16–21 (2017).
2. Lancaster, A. K., Nutter-Upham, A., Lindquist, S. & King, O. D. PLAAC: a web and command-line application to identify proteins with prion-like amino acid composition. *Bioinformatics* **30**, 2501–2502 (2014).
