# Supplementary material for: Heterotypic interactions can drive selective co-condensation of prion-like low-complexity domains of FET proteins and mammalian SWI/SNF complex
Source: Nat Commun. 2024 Feb 7;15:1168. doi: 10.1038/s41467-024-44945-5 (PMC10850361; doi:10.1038/s41467-024-44945-5)
Supplement: Supplementary file 2 — Reporting Summary [file 41467_2024_44945_MOESM2_ESM.pdf]

nature portfolio

Corresponding author(s): Priya R. Banerjee

Last updated by author(s): 12-22-2023

Reporting Summary

Nature Portfolio wishes to improve the reproducibility of the work that we publish. This form provides structure for consistency and transparency in reporting. For further information on Nature Portfolio policies, see our Editorial Policies and the Editorial Policy Checklist.

Statistics

For all statistical analyses, confirm that the following items are present in the figure legend, table legend, main text, or Methods section.

n/a	Confirmed	
☐	☒	The exact sample size (*n*) for each experimental group/condition, given as a discrete number and unit of measurement
☐	☒	A statement on whether measurements were taken from distinct samples or whether the same sample was measured repeatedly
☐	☒	The statistical test(s) used AND whether they are one- or two-sided<br>*Only common tests should be described solely by name; describe more complex techniques in the Methods section.*
☒	☐	A description of all covariates tested
☒	☐	A description of any assumptions or corrections, such as tests of normality and adjustment for multiple comparisons
☐	☒	A full description of the statistical parameters including central tendency (e.g. means) or other basic estimates (e.g. regression coefficient) AND variation (e.g. standard deviation) or associated estimates of uncertainty (e.g. confidence intervals)
☐	☒	For null hypothesis testing, the test statistic (e.g. *F*, *t*, *r*) with confidence intervals, effect sizes, degrees of freedom and *P* value noted<br>*Give P values as exact values whenever suitable.*
☒	☐	For Bayesian analysis, information on the choice of priors and Markov chain Monte Carlo settings
☒	☐	For hierarchical and complex designs, identification of the appropriate level for tests and full reporting of outcomes
☒	☐	Estimates of effect sizes (e.g. Cohen's *d*, Pearson's *r*), indicating how they were calculated

Our web collection on statistics for biologists contains articles on many of the points above.

Software and code

Policy information about availability of computer code

Data collection	ZEN (blue v2.3), VistaVision (V4.2; ISS); ZEN (SP5 2012 Black)
Data analysis	Fiji (v2.9.0) and Adobe Illustrator (v27.0) were used to prepare figures; CellProfiler 4.2.6 was used for segmentation and analysis of fluorescence microscopy images; Microsoft Excel and OriginPro (2018b) was used for graphing and statistical analysis. Scipy (v1.11.4) and Trackpy (v0.6.1) were used for the detection and tracking of optoFUS-PLD droplets.

For manuscripts utilizing custom algorithms or software that are central to the research but not yet described in published literature, software must be made available to editors and reviewers. We strongly encourage code deposition in a community repository (e.g. GitHub). See the Nature Portfolio guidelines for submitting code & software for further information.

Data

Policy information about availability of data

All manuscripts must include a data availability statement. This statement should provide the following information, where applicable:

- Accession codes, unique identifiers, or web links for publicly available datasets
- A description of any restrictions on data availability
- For clinical datasets or third party data, please ensure that the statement adheres to our policy

All data relevant to the findings of this manuscript are included in the manuscript and the supplementary appendix.

Research involving human participants, their data, or biological material

Policy information about studies with human participants or human data. See also policy information about sex, gender (identity/presentation), and sexual orientation and race, ethnicity and racism.

Reporting on sex and gender	N/A
Reporting on race, ethnicity, or other socially relevant groupings	N/A
Population characteristics	N/A
Recruitment	N/A
Ethics oversight	N/A

Note that full information on the approval of the study protocol must also be provided in the manuscript.

Field-specific reporting

Please select the one below that is the best fit for your research. If you are not sure, read the appropriate sections before making your selection.

☒ Life sciences ☐ Behavioural & social sciences ☐ Ecological, evolutionary & environmental sciences

For a reference copy of the document with all sections, see nature.com/documents/nr-reporting-summary-flat.pdf

Life sciences study design

All studies must disclose on these points even when the disclosure is negative.

Sample size	Sample sizes are chosen based upon previously optimized experimental conditions developed by the Banerjee Lab and cited in the text in appropriate sections. This was based on acquisition of statistically meaningful experimental dataset. For cellular experiments multiple cells were monitored from each biological replicate and these numbers were sufficient to make qualitative comparisons and/or statistical analysis as discussed in the manuscript.
Data exclusions	No data was excluded.
Replication	All experiments reported in this manuscript have been repeated at least two-three times with all data points shown in the relevant figures. Images and movies shown are representative of the sample and consistent with a larger set of data that was acquired.
Randomization	Randomization is not relevant to this study. Each system is homogeneous and studied individually therefore randomization is irrelevant.
Blinding	For cellular experiments on various PLD condensates, at least one of the investigators was blinded to manually identify the presence (or a lack thereof) condensates via image analysis. For other experiments, there is no information that can influence the outcome of the experiment. Parameters that are used in data acquisition are applied uniformly across all samples, which are observed in a random manner.

Reporting for specific materials, systems and methods

We require information from authors about some types of materials, experimental systems and methods used in many studies. Here, indicate whether each material, system or method listed is relevant to your study. If you are not sure if a list item applies to your research, read the appropriate section before selecting a response.

Materials & experimental systems

n/a	Involved in the study	
☒	☐	Antibodies
☐	☒	Eukaryotic cell lines
☒	☐	Palaeontology and archaeology
☒	☐	Animals and other organisms
☒	☐	Clinical data
☒	☐	Dual use research of concern
☒	☐	Plants

Methods

n/a	Involved in the study	
☒	☐	ChIP-seq
☒	☐	Flow cytometry
☒	☐	MRI-based neuroimaging

Eukaryotic cell lines

Policy information about cell lines and Sex and Gender in Research

Cell line source(s)	HEK293T cells were a kind gift from Drs. Jae and Soo Lee Lab (university at Buffalo)
Authentication	Cell line used was not authenticated
Mycoplasma contamination	Cell line used was not tested for mycoplasma contamination
Commonly misidentified lines (See ICLAC register)	No misidentified cell lines were used

Plants

Seed stocks	N/A
Novel plant genotypes	N/A
Authentication	N/A
