# Supplementary material for: Heterotypic interactions can drive selective co-condensation of prion-like low-complexity domains of FET proteins and mammalian SWI/SNF complex
Source: Nat Commun. 2024 Feb 7;15:1168. doi: 10.1038/s41467-024-44945-5 (PMC10850361; doi:10.1038/s41467-024-44945-5)
Supplement: Supplementary file 3 — Description of additional supplementary files [file 41467_2024_44945_MOESM3_ESM.docx]

**DESCRIPTION FOR ADDITIONAL SUPPLEMENTARY FILE DOCUMENT**

**Supplementary Movie 1**: HEK293T cells co-expressing OptoFUSPLD -NLS (Cry2-mCherryFUSPLD-NLS) and GFP-SS18PLD below their saturation concentrations. Upon blue light activation, OptoFUSPLD co-condenses with GFPSS18PLD . This movie corresponds to Fig. 7d. Scale bar is 10 microns.

**Supplementary Movie 2**: HEK293T cells co-expressing OptoFUSPLD -NLS (Cry2-mCherryFUSPLD-NLS) and GFP-SS18PLD. Pre-existing GFP-SS18PLD clusters act as nucleation sites for OptoFUSPLD condensate upon blue light activation. This movie corresponds to Fig. 7e. Scale bar is 10 microns.

**Supplementary Movie 3**: HEK293T cells co-expressing OptoFUSPLD -NLS (Cry2-mCherryFUSPLD-NLS) and GFP-SS18PLD. Pre-existing GFP-SS18PLD condensates serve as seeds to nucleate OptoFUSPLD-NLS condensates upon blue light activation. This movie corresponds to Fig. S18. Scale bar is 10 microns.
